# Gain time to adapt: How sorghum acquires tolerance to salinity

**DOI:** 10.3389/fpls.2022.1008172

**Published:** 2022-10-17

**Authors:** Eman Abuslima, Adnan Kanbar, Manish L. Raorane, Elisabeth Eiche, Björn H. Junker, Bettina Hause, Michael Riemann, Peter Nick

**Affiliations:** ^1^ Molecular Cell Biology, Botanical Institute, Karlsruhe Institute of Technology, Karlsruhe, Germany; ^2^ Department of Botany, Faculty of Science, Suez Canal University, Ismailia, Egypt; ^3^ Institute of Pharmacy, Martin-Luther-University, Halle-Wittenberg, Halle, Germany; ^4^ Institute of Applied Geosciences, Karlsruhe Institute of Technology, Karlsruhe, Germany; ^5^ Laboratory for Environmental and Raw Materials Analysis (LERA), Karlsruhe Institute of Technology, Karlsruhe, Germany; ^6^ Cell and Metabolic Biology, Leibniz Institute of Plant Biochemistry (IPB), Halle, Germany

**Keywords:** salt stress, sorghum, sodium transport, sucrose, proline, redox homeostasis

## Abstract

Salinity is a global environmental threat to agricultural production and food security around the world. To delineate salt-induced damage from adaption events we analysed a pair of sorghum genotypes which are contrasting in their response to salt stress with respect to physiological, cellular, metabolomic, and transcriptional responses. We find that the salt-tolerant genotype Della can delay the transfer of sodium from the root to the shoot, more swiftly deploy accumulation of proline and antioxidants in the leaves and transfer more sucrose to the root as compared to its susceptible counterpart Razinieh. Instead Razinieh shows metabolic indicators for a higher extent photorespiration under salt stress. Following sodium accumulation by a fluorescent dye in the different regions of the root, we find that Della can sequester sodium in the vacuoles of the distal elongation zone. The timing of the adaptive responses in Della leaves indicates a rapid systemic signal from the roots that is travelling faster than sodium itself. We arrive at a model where resistance and susceptibility are mainly a matter of temporal patterns in signalling.

## Introduction

Salt stress is to be considered as one of the major abiotic stresses with a very negative ecological impact on crop productivity. Salinised soils exceed 4 dS m^−1^ (≈ 40 mM) of NaCl (Chinnusamy et al., 2005). The impact of salt stress is progressively accentuated for several reasons. First, global warming causes rising sea levels, such that seawater penetrates into coastline farmlands. Second, anthropogenic activities, such as damming, cause fluctuation in sediment transport into the downstream plains which results in salinisation of coastline aquifers (Hzami et al., 2021). Third, artificial irrigation with low-quality water will leave salt upon evaporation (Shani and Dudley, 2001). These three factors are drivers of rising salinity that turns fertile agricultural areas into marginal lands that slip out of agricultural use. Crops able to cope with these harsh conditions would allow farmers to find alternative sources of income.

In this context, sorghum (*Sorghum bicolor* L.) has attracted attention, since it copes better with salinity than other cereal crops (Boursier et al., 1987). Sorghum can, thus, adjust to the conditions of marginal lands, such as those along the coastline. Among cereal crops, it ranks on position five with respect to economic impact, due to versatile usage including human nutrition and animal feed. These traditional uses have been complemented recently by a progressive role of sorghum for bioeconomy. As a C_4_ plant, sorghum can minimise resource losses due to photorespiration, and consequently grows extremely rapidly, linked with an intense mass translocation of sucrose from the leaves into the stem (Bihmidine et al., 2015; Kanbar et al., 2021a). Therefore, among cereal crops, sorghum is considered as one of the plants with the greatest potential to produce bioethanol (Irving, 2015). The use of agricultural land for the production of bioenergy is progressively seen critically (Thompson, 2012). With its pronounced stress-resilience, multiple use, and the ability to grow on marginal lands, sorghum would allow to circumvent the “no food for fuel” dilemma.

Salt stress causes major drawbacks in plant growth because of two harmful components: (i) osmotic stress, and (ii) ionic toxicity of Na^+^ and Cl^-^. The response to the osmotic component is rapid and immediate, owing to the negative water potential in the soil surrounding the roots. The resulting water loss leads to stomatal closure and a swift drop in the rate of transpiration (Munns and Tester, 2008). The ionic component has a slower effect that ensues after Na^+^ had reached the photosynthetic tissues, where it interferes with electron transport across the thylakoid leading to the production of Reactive Oxygen Species (ROS), bleaching of chlorophyll with a negative impact upon photosynthesis (Zhang et al., 2014). Also, chloride can cause damage by still unclear mechanisms (Isayenkov and Maathuis, 2019).

Salinity has shaped plant evolution from its very beginnings favouring adaptive mechanisms, such as avoiding ionic stress, controlling the distribution of ions through reduced Na^+^ uptake by roots (Jiang et al., 2019)*;* extrusion of Na^+^ to the apoplast; reduced transition of Na^+^ into the transpirational stream, and sequestering Na^+^ in the vacuole, to retain it in specific tissues while maintaining turgescence. Alternatively, cells can adapt to ionic stress, for instance by synthesis of compatible osmolytes that will mitigate the gradient in water potential to the rhizosphere, or by deploying enzymatic and non-enzymatic antioxidants to restore redox homeostasis (Hoque et al., 2008; Nahar et al., 2016; Wu et al., 2019). Under certain circumstances, cells under the challenge of salinity can turn to programmed cell death rather than investing resources for local adaptation. While deleterious to the cell itself, this may be beneficial for the plant as an entity because resource can be allocated to the meristematic tissues that allow for regeneration, once the stress episode is over (Li et al., 2007). In some cases, the dead organs can be shed, thus, removing also the ions accumulated in them.

Avoidance of ionic stress targets sodium transport and compartmentalisation (for review see Schroeder et al., 2013). The accumulation of Na^+^ ions around the roots facilitates Na^+^ efflux into root cells mainly by non-selective cation channels (NSCC) along the electrochemical gradient. Using the plasma membrane Na^+^/H^+^ antiporter protein SOS1 the cell exports sodium back to the apoplast, i.e., against the concentration gradient. Furthermore, the vacuolar Na^+^/H^+^ antiporter NHX1 sequesters Na^+^ to the vacuole which will not only restore Na^+^/K^+^ homeostasis in the cytosol, but also allows the cell to remain turgescent and to sustain expansion, even though the water potential of the environment is very negative. Ions transported into roots will be transferred to shoots through the transpiration stream after xylem loading. The control of Na^+^ transfer to shoots is considered an important trait in salt tolerant plants and involves a membrane passage at the endodermis. In addition, the subfamily I of HKT transporters plays a vital role in this process as it resides at the plasma membrane of root xylem parenchyma cells and can prevent sodium from reaching the shoot by retrieving Na^+^ from xylem vessels (for reviews, see Almeida et al., 2013; Keisham et al., 2018).

Avoidance of ionic stress is often complemented by adaptation to the consequences of ionic stress. As to be effective, this adaptation must be under control by signals. To understand, how plants sense, signal and adapt to salt stress is of major priority for breeding programs. During the early stress phase, the mutual timing of early signalling events decides about the ensuing type of response – either cellular adaption or programmed cell death for the sake of the whole plant)for reviews, see Ismail et al., 2014a; Riemann et al., 2015). Sodium enters by NSCCs, while osmotic water loss triggers mechanosensitive calcium channels. The entry of calcium is accompanied by proton influx *via* co-transport increasing the alkalinity of the apoplast which stabilises the apoplastic ROS generated *via* the Ca^+2^-activated NADPH oxidase (Knight et al., 1997; Tester and Davenport, 2003; Donaldson et al., 2004; Ismail et al., 2012; Wolf et al., 2012). Downstream events include the accumulation of phytohormones, mainly jasmonates (JAs) and abscisic acid (ABA). For instance, both hormones control stomatal closure as one of the earliest systemic adaptations when roots are confronted with salinity (Suhita et al., 2004).

The ability to cope with salinity stress correlates with the swiftness and amplitude of ABA accumulation (Ismail et al., 2014b) improving osmotolerance through LEA proteins or proline (for reviews see Joshi-Saha et al., 2011; Banerjee and Roychoudhury, 2017; Zarattini and Forlani, 2017). The accumulation of ABA is controlled by jasmonic acid at several levels, especially under salinity (reviewed in Ismail et al., 2014b). While JA is certainly not the only upstream regulator of ABA accumulation (at least with respect to osmotic stress), the decision between adaption and programmed cell death in response to salinity is depending on the temporal signature of JA signalling. A rapid, but transient signature is followed by efficient accumulation of ABA triggering protective events that lead to cellular adaptation, while a sluggish, but persistent signature of JA signalling culminates in cell death (reviewed in Ismail et al., 2014b). As proof of concept for this signature model, rice was engineered with a tailored version of the JA signalling protein JAZ8 that is dominant-negative with respect to proteolysis, enforcing a transient JA signature. In fact, these transgenic plants exhibited elevated salinity tolerance (Peethambaran et al., 2018).

Thus, adaptation to salt stress is composed of many layers that must be orchestrated in time. Understanding the role of a given molecular event in this finely tuned network needs the functional context. Moreover, it is not self-evident, whether a given event is of adaptive nature or whether it is just a manifestation of stress-related damage. An efficient way to sort damage related from adaptive events has been the use of contrasting pairs of otherwise closely related experimental systems, such as a wild type versus a mutant in the same wild-type background (Hazman et al., 2015), or two closely related species that differ in their stress response (Ismail et al., 2012). In the current study, we use the same strategy, making use of a standardised hydroponic system to ensure the comparability of conditions. We investigated a pair of sorghum genotypes differing in salinity tolerance. Della, a sweet sorghum versus Razinieh, a grain sorghum had already been mapped in our previous study and found to contrast with respect to their response to P_i_ depletion with superiority to Razinieh (Kanbar et al., 2021b). However, the current study shows that Della is more tolerant to salt stress, which is reflected by a more favourable ion uptake pattern, but also by a more robust oxidative homeostasis. We employ transcript quantification by qRT-PCR and fluorescent labelling of Na^+^ in the roots to show that the reduced transfer of Na^+^ to and the superior K^+^ retention in the shoot in Della is orchestrated at both, transcriptional and cell physiological levels. In parallel, a swifter activation of hormonal signalling, amino acids, and antioxidant activity supports the notion that it is the temporal coordination of the early salinity response that helps to acquire adaption to salinity.

## Materials and methods

### Plant materials, cultivation, and stress treatments

The study used two varieties of *Sorghum bicolor* (L.) Moench, the grain sorghum ‘Razinieh’, and the sweet sorghum ‘Della’. Razinieh is an improved landrace from Syria (Kanbar et al., 2020), while Della derives from a cross of Dale and ATx622 developed in the Virginia Polytechnic Institute (McKinley et al., 2016). The caryopses were sown in plastic boxes (Magenta, Sigma-Aldrich) containing 8% MS medium solidified with 0.5% phytoagar (Duchefa, Netherlands). Seedlings were grown for 10 days in a culture room with a 12 h photoperiod at 120 μmol. m^–2^s^-1^ light intensity at 25°C and 12 h in darkness at 22°C. Upon selection for uniformity and size, seedlings were transferred subsequently to hydroponic cultures. These consisted of custom-made sterilised floating racks in a glass jar containing milli-pore water enriched with half-strength (2.15 g/L) MS basal salt mixture (standard culture solution). After transfer to the hydroponic culture all seedlings adapted for 3 days in the standard culture solution before starting salt (half strength MS + 100 mM NaCl) or control (standard culture solution) treatments, respectively. The 13 days old seedlings were studied under these treatments for 12 days. The sampling times were determined for each experiment.The leaves and roots of control and stressed plants were harvested, frozen in liquid nitrogen, and stored at -80°C to be used for biochemical and molecular analyses.

### Phenotypic analysis

To determine biomass, shoots and roots were excised by a sterilised razor blade upon harvesting, after 1, 3, 6, 9, and 12 days from stress treatment, and then oven-dried at 48°C for 2 days till constant dry weight. Leaf area was quantified from digital images using quantitative image analysis (ImageJ, https://imagej.nih.gov/ij/). Relative Water Content (RWC) was determined as described in Tang et al. (2020), chlorophyll was extracted and quantified according to Metzner et al. (1965). Malondialdehyde (MDA) as readout for lipid peroxidation was measured using the thiobarbituric acid (TBA) method (Heath & Packer, 1968), and hydrogen peroxide (H_2_O_2_) content was measured using potassium buffer pH 7 and 1 M KI according to Shi et al. (2005). Data represent mean values from three independent biological replicates.

### Determination of Na^+^ and K^+^ content

Oven-dried samples (at 48°C for 2 days, ~50 mg) from each treatment were ground to a fine powder (TissueLyser, Qiagen) and placed in 50 ml digestion tubes (Gerhardt, UK). Subsequently, 0.5 mL ultrapure water, 2 mL HNO_3_ (65% v/v, heated) and 0.5 mL H_2_O_2_ (30% v/v, p.A.) were added to the samples, prior to incubation in a heating block (DigiPrep jr, S-prep) system at 110°C for 2-3 h, before evaporation to near dryness. The final volume of each sample was adjusted to 20 mL with 1% v/v HNO_3_. To check the quality of the digestion procedure, blank samples and two reference materials (tomato leaves - NIST 1573a; grass - 14^th^ needle/leaf interlaboratory test) were included into the process. Sodium and potassium were quantified by Inductively Coupled Plasma Optical Emission Spectrometry (ICP-OES, 715ES, Varian, radial mode*)*. The stability of the measurement was checked by regularly measuring two calibration standards and variance was ≤ ± 3.3%. Each treatment was sampled in 3 biological replicates. Blank samples were subjected to digestion and measurement in the same way, just omitting the plant sample.

### Quantification of redox homeostasis

Methanolic extracts of freeze-dried sorghum shoots were assayed for scavenging activity against the 2,2-diphenyl-1-picrylhydrazyl radical (DPPH) (Alara et al., 2018) and 2,2′-azino-bis(3-ethylbenzothiazoline-6-sulfonate) (ABTS) (Nenadis et al., 2004). Butylated hydroxyanisole (BHA), a very efficient antioxidant, served as positive control in both assays. Total phenolics content was determined with the Folin-Ciocalteu method (Singleton and Rossi, 1965) from 100 µl of the methanolic crude extract (~10 mg/ml) as ferulic acid equivalents (mg FA/g DW), along with aluminum chloride colorimetry (Zhishen et al., 1999) expressed as quercetin equivalents (mg QA/g DW). Superoxide Dismutase (SOD) activity was assayed by monitoring the inhibition of the photochemical reduction of Nitroblue Tetrazolium (NBT) at 560 nm (Beauchamp and Fridovich, 1971), Ascorbate Peroxidase (APX) was assayed following Nakano and Asada (1981), general Peroxidase activity (POX) according to Malik and Singh (1980), Catalase (CAT) activity following the method of Aebi (1974), and Glutathione Reductase (GR) according to Venisse et al. (2001). All enzyme activities were determined in biological triplicates following quantification of total protein content according to Bradford (1976).

### RNA extraction and quantitative real-time PCR

Total RNA was isolated from the 2nd leaves and roots of control and salt stressed plants at defined time points using the InnuPrep plant RNA kit (Analytika Jena RNA kit) according to the manufacturer instructions. Reverse transcription into cDNA and quantitative real-time PCR were conducted as described in Hazman et al. (2015), using *Ubiquitin* (*SbUBQ*) as an internal standard. The transcript levels between the different samples were compared using the 2^−ΔCt^ method (Livak and Schmittgen, 2001). Data represents mean and standard error from three independent biological replicates (each biological replicate in three technical replicates). The details of the oligonucleotide primers to amplify the genes of interest are provided in **Table S1**.

### Metabolite analysis

Total metabolites were extracted as described earlier (Gemmer et al., 2020) with few modifications from lyophilised and ground material. Soluble sugars were determined by following an optimized method based on GC-MS (GC-Triple Quadrupole system - 7890A/5975C/Chromtech Evolution 3, Agilent, Santa Clara, USA). Total free amino acids were quantified by HPLC-FLD after derivatisation with *o*-phthalaldehyde (and 9-fluorenylmethyl chloroformate (FMOC) as described in the instruction manual of the producer (1260 Infinity II amino-acid solution, Agilent, Santa Clara, CA, USA). For detailed description on sugar and amino acid analytics, please see Supplementary method file.

### Quantification of endogenous hormones

The contents of jasmonic acid (JA), its bioactive isoleucine conjugate (JA-Ile), its pre-cursor 12-oxophytodienoic acid (OPDA), and of abscisic acid (ABA) were simultaneously quantified at 0, 1, 6, and 12 h of the stress treatment, following a standardised method based on ultraperformance liquid chromatography tandem mass spectrometry (UPLC-MS/MS) according to (Balcke et al., 2012) using [^2^H_5_]OPDA, [^2^H_6_]JA, [^2^H_2_]JA-Ile, and [^2^H_6_] ABA as internal standards. Data represent mean and standard errors from three independent biological replicates.

### Visualisation of vacuolar and cytosolic Na^+^ distribution in different root zones

The distribution and accumulation of sodium ions was visualised using the fluorescent dye CoroNa Green acetoxymethyl ester (C36676, Invitrogen) according to Wu et al. (2018). In brief, four-day-old sorghum seedlings (grown on solid agar medium in darkness) were transferred to hydroponic culture containing 100 mM NaCl for 24 h. Two segments of 10 mm length were excised from seminal sorghum roots—one in the maturation zone (defined by the presence of root hairs, 30–40 mm from the apex), the other in the apex (the first 10 mm). To visualise sodium in relation to the plasma membrane, the root segments were simultaneously stained with 20 μM CoroNa Green-AM and 20 μM FM4-64 (Molecular Probes) for 2 hours in darkness. The samples were then rinsed with 5 mM MES buffer, pH 6.3, adjusted with KOH, to remove unbound dye, and then directly analysed under an AxioObserver Z1 inverted microscope (Zeiss, Jena, Germany) equipped with a spinning disc device (Yokogawa CSU-X1 Spinning Disc Unit, Yokogawa Electric Corporation, Tokyo, Japan). Images were recorded using the 488 nm (CoroNa) and the 509 nm (FM4-64) lines of the Ar-Kr laser for excitation and a Plan-Apochromat 25x/1.44 DIC oil objective operated *via* the Zen 2012 (Blue edition, Zeiss) software. Mean fluorescence intensity probed in cytoplasm and vacuole were quantified (ImageJ, https://imagej.nih.gov/ij/) for each cell from images that had been recorded with constant exposure time and laser power to ensure comparability. Data represent averages and standard errors from 53–98 individual cells for each genotype over 3 independent biological replicates.

### Visualisation of Casparian strips

Sorghum seedlings were raised under standard conditions (as described in the first section of the method part). Root samples were collected after 6 days of salt stress from both control and salt-treated seedlings. Roots were excised from the distal maturation zone (15 mm from the root tip) and fixed for 30 min in fixative (4% w/v paraformaldehyde and 3% v/v glutaraldehyde in 0.025 M sodium phosphate buffer), followed by three washings with 0.025 M sodium phosphate buffer alone. Subsequently, the samples were dehydrated through a rising ethanol series, and eventually embedded in paraffin wax (Paraffin 52-54°C, Carl Roth GmbH; Germany). Cross sections of 15 µm thickness were cut by a microtome (Jung, Heidelberg), and stained according to Brundrett et al. (1988). Stained sections were viewed with a Zeiss Axioskop FS Fluorescence Microscope using blue light (filters: excitation 450–490 nm, dichroic mirror 510 nm, emission LP 520). Each treatment was conducted in three independent biological replicates.

### Statistical analysis

The statistical significance of mean values of different treatments was performed by Student’s *t*-test (*P< 0.05, **P <0.01, ***P< 0.001, ****P< 0.0001) or Duncan’s test, with a significance level of *P* ≤ 0.05 using software IBM SPSS Statistics 22. All data were collected from the mean of at least three independent biological replicates.

## Results

### Under salt stress, Della partitions less sodium to the shoot in comparison to Razinieh

**Figure 1:**
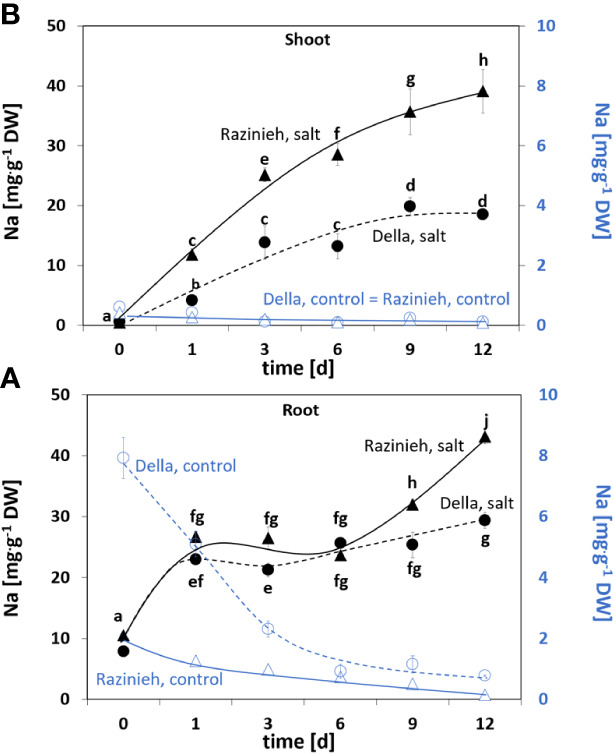
Content of sodium ions in **(A)** roots and **(B)** shoots of sorghum genotypes Della and Razinieh. Thirteen days old Della (dashed line), and Razinieh (solid line) seedlings were stressed in aqueous NaCl (100 mM) solution for 1, 3, 6, 9, and 12 days. Values represent the mean of at least three independent biological replicates ± SE. Different letters show significant differences between different genotypes and treatments according to Duncan’s test (*P *< 0.05).

To see potential differences in Na^+^ uptake and re-distribution, we followed Na^+^ content in roots and shoots of Della and Razinieh under salt stress and observed three distinct stages of Na^+^ uptake into the roots (**Figure 1A**):

(i) During the first day, sodium content of the roots increased rapidly to around threefold of the initial value. During this “initial phase” of stress challenge, both genotypes behaved equally. (ii) During the subsequent five days, sodium content kept a plateau, indicating that during this “decision phase” sodium uptake from the medium and sodium transfer to the shoot were in a dynamic equilibrium. (iii) Eventually, the roots entered a “manifestation phase”, where the two genotypes became progressively different. While in Della the plateau was kept, the Na^+^ content in Razinieh increased further at days 9 and 12.

In contrast, the situation in the shoot is clearly different from the very beginning (**Figure 1B**). Here, sodium content increased in Razinieh twice as fast during the first three days compared to Della. Again, there was an intermittent “decision phase” between days 3 and 6. Here, Della kept a plateau, while Razinieh increased further as compared to the initial increase. Following day 6, the values rose only moderately for Della, and even came to a halt at day 9, while in Razinieh the increase of sodium in the shoot proceeded unrestrained. We estimated the coefficient for transfer from root to shoot by a simple mathematical model based on the steady state in the “decision phase”, such that the time constants could be inferred (**Supplementary Figure S1**). The estimated transfer coefficient was 7 times higher in Razinieh than in Della.

Since sodium stress often impairs potassium homeostasis, we also assessed potassium content of the same plants. We found a significant (*P*<0.05) depletion of potassium from roots of both genotypes (**Supplementary Figure S2B**), such that the ratio of potassium over sodium was dropping from 6.50± 0.17 (Della), or 4.17 ± 0.12 (Razinieh) to <2 already after the first day of salt stress in both genotypes (**Supplementary Figure S2D**). In contrast with the shoots, in Della, potassium content was mostly sustained under salt stress comparing to the controls (**Supplementary Figure S2A**), while in Razinieh, the potassium content remained at the initial level (0 day). Again, as a result of sodium uptake, the ratio of potassium over sodium dropped drastically in both genotypes (**Supplementary Figure S2C**). While the initial value was 75.90 ± 15.60 for Della and 90.13 ± 6.30 for Razinieh, the value had decreased to around 10 in Della and even to 2.90 for Razinieh after the first day of salt stress. Also, during the subsequent days, the values for Razinieh were significantly lower than those seen for Della.

### Leaves of Della cope better with salt stress

To see how the physiology would change during the “decision phase”, sorghum seedlings were subjected to 100 mM NaCl and sampled at days 1, 3, and 6. As readout, morphology, coverage of green area, chlorophyll content, and Relative Water Content (RWC) were scored for the second leaf (**Supplementary Figure S3**).

All these parameters showed that Razinieh was more susceptible to salt stress. At day 6 of salt exposure, the leaves of Della did not display any difference from the controls (**Supplementary Figure S3A**), while the leaves of Razinieh showed wilting and necrosis. This was also reflected by reduced coverage of green area in the second leaf in Razinieh, while the value remained stable in Della (**Supplementary Figure S3B**). The higher susceptibility of Razinieh was also manifest by a reduction of chlorophyll content compared to the control (**Supplementary Figure S3C**). The decline in chlorophyll content and green area was followed by a significant drop of RWC (**Supplementary Figure S3D**), which accelerated to around ¼ of the control value at day 6. Consequently, root and shoot dry weight decreased massively in Razinieh. Again, no such decrease was noted in Della (**Supplementary Figures S4A, B**).

### Leaves of Della show more efficient redox homeostasis

**Figure 2:**
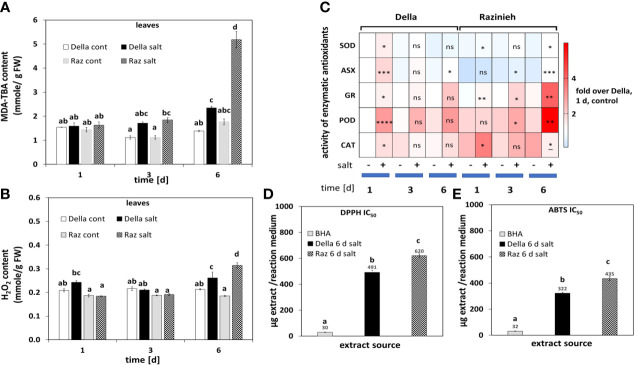
**(A)** The level of malondialdehyde (MDA) was estimated in the 2^nd^ leaves of control and salt-stressed Della and Razinieh seedlings. **(B)** Levels of aqueous peroxide in the 2^nd^ leaves of control and salt-stressed Della and Razinieh seedlings. **(C)** Heat map profile of enzymatic antioxidants in 2^nd^ leaves of both Della and Razinieh under salinity stress compared to the control of Della at day 1. **(D)** IC50 values were calculated from DPPH free radical scavenging activity of 6 days stressed shoots depending on regression analysis in **Supplementary Figure S6A**. **(E)** IC50 values were calculated from ABTS free radical scavenging activity of 6 days stressed shoots depending on regression analysis in **Supplementary Figure S6B**. Values represent the mean of at least three independent biological replicates ± SE. Different letters show significant differences between different genotypes and treatments according to Duncan’s test (*P*<0.05). Asterisks indicate a statistically significant difference between treatment and corresponding control, as determined by Student’s *t*-test (*P< 0.05, **p < 0.01, ***P < 0.001, ****P < 0.0001: * significant decrease, and ns non-significant).

To address the ability for ROS scavenging, we measured lipid peroxidation as readout for oxidative damage, hydrogen peroxide content as most stable ROS, as well as the performance of enzymatic and non-enzymatic antioxidative systems as readout of redox balance. In both genotypes the level of MDA was mildly, but significantly elevated from day 3 of salt stress. In Razinieh there was a three-fold increase at day 6 (**Figure 2A**), paralleled by 41% increase in the steady-state level of H_2_O_2_, compared to only 23% in Della (**Figure 2B**). In summary, these data show that Della can constrain salt-induced ROS better than Razinieh.

While both genotypes significantly increased the activity of enzymatic scavengers under salinity (**Figure 2C**), there were specific differences in the amplitude and the quality of the response. Della showed a rapid, but transient, activation of Superoxide Dismutase (SOD) and Ascorbate Peroxidase (ASX), while Razinieh showed late and minor activation of both enzymes. Also, Peroxidases (POD) were activated in Della more vigorously, but were delayed in Razinieh, overshooting at day 6, correlating with the accumulation of phenolics (potential substrates for peroxidases). This is not seen during the early increase of POD activity in Della (**Supplementary Figure S5A**), indicating that the functional context of these POD activities differs. However, Glutathion Reductase (GR), and Catalase (CAT) activities followed a contrasting pattern. Both genotypes steadily increased GR activity, with significantly higher levels in Razinieh. For CAT, a transient peak at day 1 (more distinct in Razinieh), was followed by a decline till day 6 in both genotypes. In summary, a rapid and transient activation of SOD, ASX and POD correlate with salt tolerance, while elevated GR and POD at later stress stages seem to be markers for stress damage. Scavenging of the synthetic free radicals ABTS and DPPH as readout for non-enzymatic antioxidants (**Supplementary Figures S6A, B**) show high activity with lower IC_50_ values in Della (by 20-25%) compared to Razinieh (**Figures 2D, E** **)**, meaning that the late activation of POD in Razinieh **(** **Figure 2C** **)** was not accompanied by an equivalent response of non-enzymatic antioxidants.

### Salt tolerance of Della correlates with earlier accumulation of sugars and proline in the leaves

**Figure 3:**
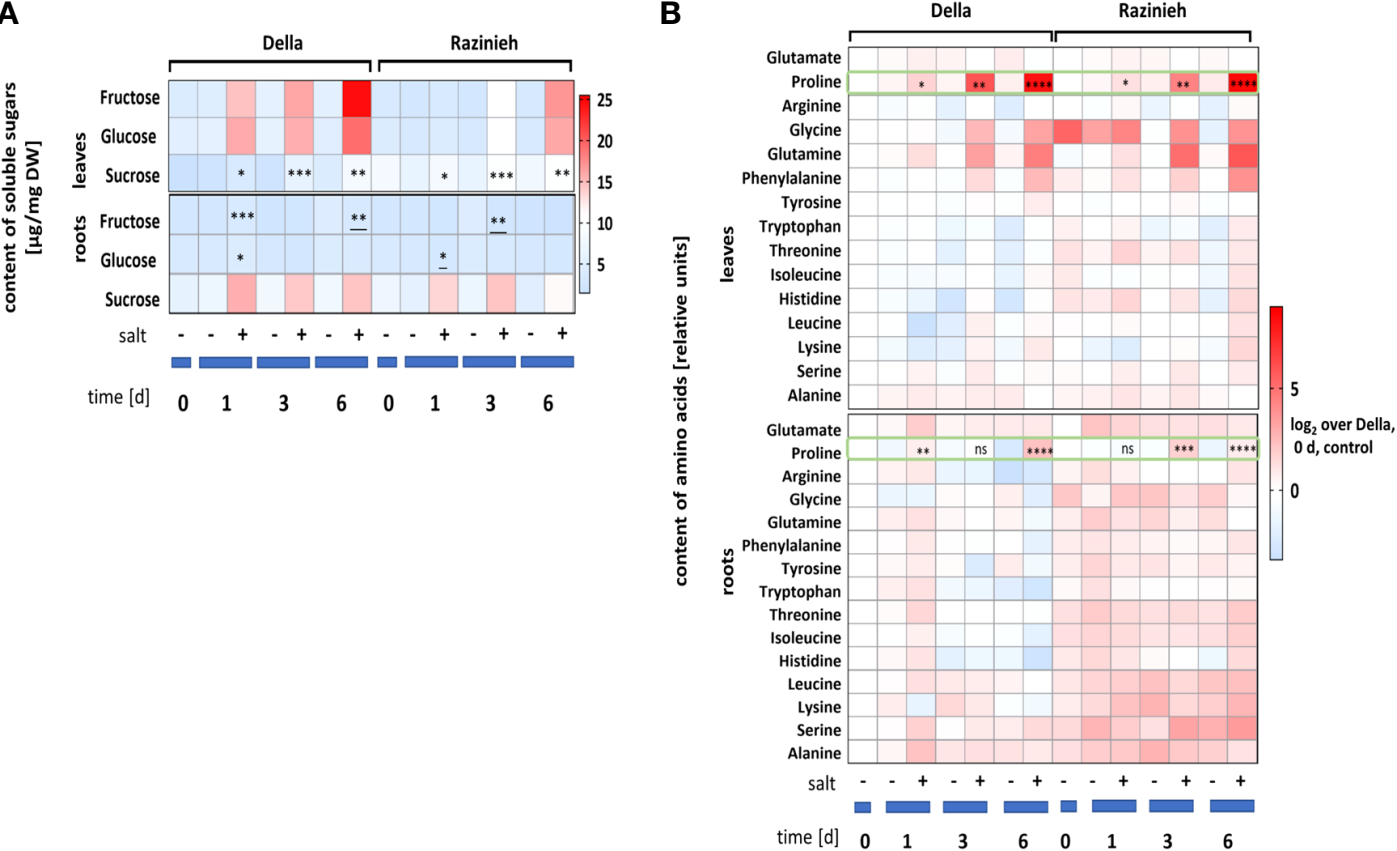
Metabolites abundance estimated in roots and 2^nd^ leaves in control and salt-stressed Della and Razinieh thirteen days old seedlings stressed in aqueous NaCl (100 mM) solution for 1, 3, 6 days. **(A)** absolute values of soluble sugars: glucose, fructose, and sucrose. **(B)** Log_2_ ratios of mean amino acids abundance compared to the control of Della at day 0. Values represent the mean of at least three independent experiments ± SE. Asterisks indicate a statistically significant difference between treatment and corresponding control, as determined by Student’s *t*-test (*P < 0.05, **p < 0.01, ***P < 0.001, ****P < 0.0001: * significant decrease, and ns non-significant).

To understand, why Della leaves can buffer oxidative stress, we followed the salinity responses of sugars (**Figure 3A**), and amino acids (**Figure 3B**). In most cases, this response was more pronounced in the leaves compared to the roots. The most salient response was the rapid and strong accumulation of fructose and glucose in the leaves of Della, which was much slower and far less evident in leaves of Razinieh (**Figure 3A**). For roots, sucrose accumulated more swiftly and persistently in Della (125% at day 1) as compared to Razinieh (85% at day 1). The situation in the leaves was different with twice the sucrose content in Razinieh over Della for both, control, and salt treatment. However, the steady-state levels for sucrose increased under salt stress, slightly, but significantly, in both genotypes. The most striking response of amino acids, was a rapid and strong accumulation of proline in the leaves (more vigorously in Della), which was barely detectable in the roots (**Figure 3B**). Generally, most amino acids were elevated in Razinieh over those seen in Della, for any condition. This was especially evident for glycine and glutamine in the leaves which were also more pronounced in Razinieh. Both phenomena were barely detectable in the roots.

### Salt tolerance of Della correlates with vacuolar Na^+^ sequestration in the root elongation zone

**Figure 4:**
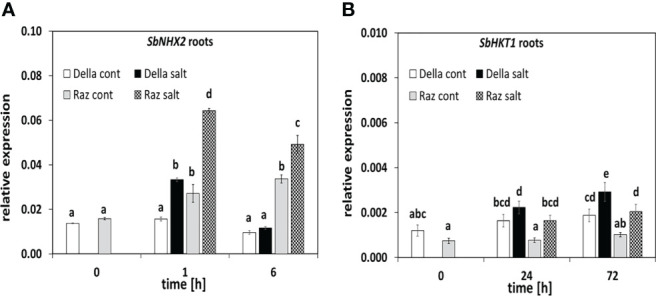
The steady-state transcripts level of salt stress-related genes in sorghum roots. Thirteen days old Della (white bars), and Razinieh (gray bars) seedlings were stressed in aqueous NaCl (100 mM) solution to measure steady sate transcripts of **(A)** *SbNHX2* after 1, and 6 h and **(B)** *SbHKT1* after 24 h and 72 h after salt treatment. Values represent the mean of at least three independent biological replicates ± SE. Different letters show significant differences between different genotypes and treatments according to Duncan’s test (*P *< 0.05).

To understand the mechanisms behind the reduced sodium transfer to the shoot in Della (**Figure 1B**), we followed the expression of the central sodium transporters, *SbSOS1* and *SbNHX2* (**Figure 4A**). The expression level of *SbSOS1* as main sodium exporter was around one order of magnitude lower in the root as compared to the leaf (**Supplementary Figures S7A, B**), already indicating that this exporter does not play a major role in the root. The ground level of *SbSOS1* in the root was less than half in Razinieh as compared to Della (**Supplementary Figure S7B**), and despite some increase during root development, it did not reach the level seen in Della. The situation in the leaf was contrasting (**Supplementary Figure S7A**). Here, the ground transcript levels of *SbSOS1* were higher in Razinieh (by a factor of 2.5) compared to those in Della. Under salinity, they were transiently suppressed in both genotypes, but recovered later. In the summary, a higher *SbSOS1* ground levels in the root of Della correlated with lower levels of *SbSOS1* in the leaves, while the pattern was reversed for Razinieh. The inducibility by salt appeared to be inversely related with the initial steady-state level of this transcript.

For the Na^+^/H^+^ vacuolar antiporter *SbNHX2*, the ground levels were similar in roots of both genotypes. In response to salt stress, this transcript was rapidly (1 h), but transiently induced. This induction was stronger in Razinieh leading to around twice the steady-state level of that seen in Della after 1 h (**Figure 4A**), while in the leaves, none of the genotypes showed significant changes, although, like for *SbSOS1*, both showed higher transcripts levels compared to the root.

Among alternative transporters that withhold sodium in the root, we measured transcript level for the high-affinity potassium transporter *SbHKT1* that were significantly higher in Della (**Figure 4B**). While this transcript was induced in both genotypes, the levels reached in Razinieh in response to salt stress were just approaching the levels seen in Della prior to induction. Thus, the more active expression of this type of transporters might play a role in the reduced root-to-shoot transfer of sodium (**Figure 1B**).

**Figure 5:**
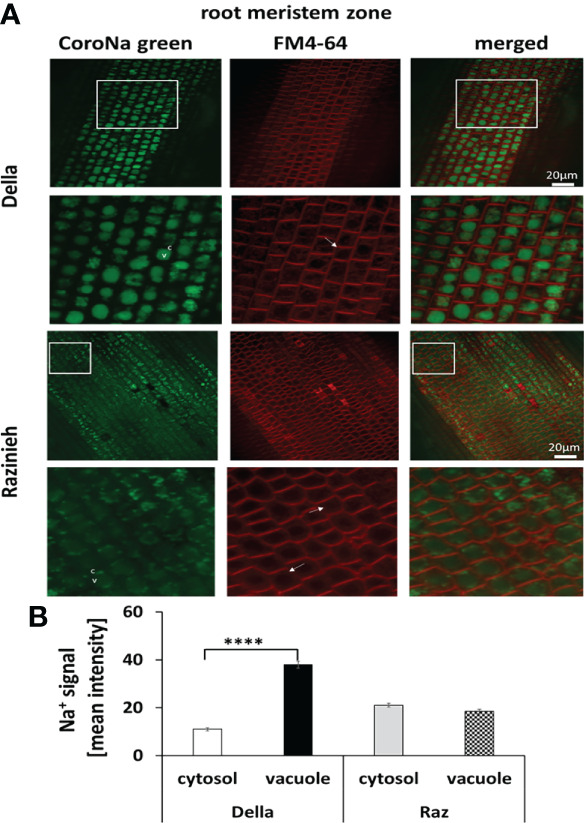
Na^+^ signal intensity in the root meristem zone of 4-day-old sorghum seedlings grown in darkness treated with 100 mM NaCl for 24 h **(A)** Representative images of root meristem zone cells of Della and Razinieh genotypes stained with CoroNa Green dye, and FM4- 64 dye **(B)** Averaged values for Na^+^ signal intensity in the cytosol and vacuole in the root meristem zone. Mean ± SE [*n *= 77 (Della) and 57 (Razinieh)]. Asterisks indicate a statistically significant difference between treatment and corresponding control, as determined by Student’s *t*-test (****P < 0.0001).

**Figure 6:**
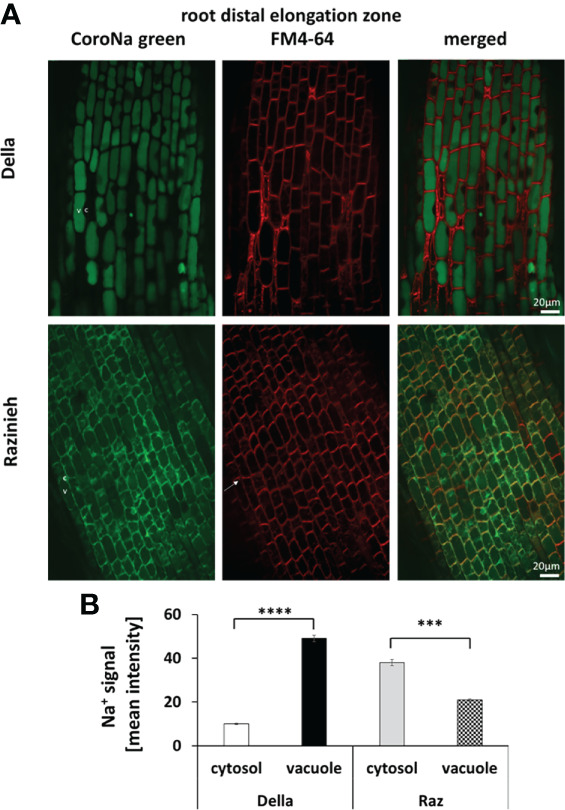
Na^+^ signal intensity in the cytosol and vacuole in the root distal elongation zone of 4-day-old sorghum seedlings grown in darkness were treated with 100 mM NaCl for 24 h **(A)** Representative images of root distal elongation zone of Della and Razinieh genotypes, cells stained with CoroNa Green dye, and FM4- 64 dye. **(B)** Averaged values for Na^+^ signal intensity in the cytosol and vacuole in the root distal elongation zone. Mean ± SE [*n *= 98 (Della) and 53 (Razinieh)]. Asterisks indicate a statistically significant difference between treatment and corresponding control, as determined by Student’s *t*-test (***p < 0.001, ****P < 0.0001).

**Figure 7:**
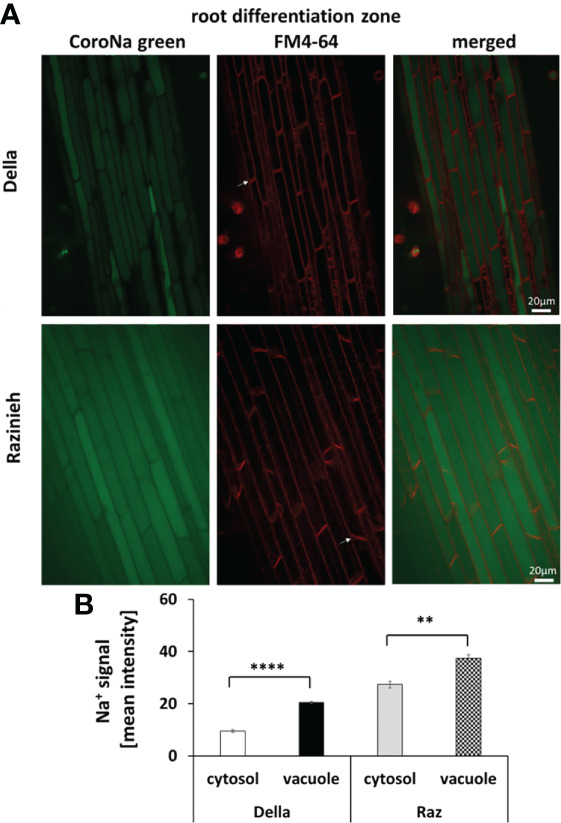
Na^+^ signal intensity in the cytosol and vacuole in the root differentiation zone of 4-day-old sorghum seedlings grown in darkness were treated with 100 mM NaCl for 24 (h) **(A)** Representative images of root differentiation zone of Della and Razinieh genotypes, cells stained with CoroNa Green dye, and FM4- 64 dye. **(B)** Averaged pooled values for Na^+^ signal intensity in the cytosol and vacuole in the root differentiation zone. Mean ± SE [*n* = 37 (Della) and 47 (Razinieh)]. Asterisks indicate a statistically significant difference between treatment and corresponding control, as determined by Student’s *t*-test (**p < 0.01, ****P < 0.0001).

To understand the complex patterns of the two sodium transporter transcripts, we investigated the actual distribution of sodium in different root zones, using a double-staining with FM4-64 (labelling the plasma membrane) and the fluorescent sodium dye CoroNa Green followed by spinning disc confocal laser-scanning microscopy. In general, Della was more efficiently sequestering Na^+^ in the vacuole than Razinieh (**Figures 5–7**). In the meristematic zone of Della, the vacuolar sodium signal was 2.5-fold higher as compared to cytoplasm (a mean intensity of 37.9 ± 1.5 versus 10.9 ± 0.7, *P*<0.01). In contrast, there was hardly any difference in Na^+^ signals between vacuole and cytoplasm for Razinieh (18.5 ± 0.9 versus 21.1 ± 0.8; *P*<0.05), indicating that sodium was not sequestered here, but redistributed homogenously (**Figure 5**).

A major, qualitative, difference between the two genotypes was observed for the distal elongation zone of the root (**Figure 6**). Here, Della successfully managed to sequester Na^+^ in the vacuole by 5-fold over the signal seen in the cytoplasm (49.0 ± 1.5 versus 9.9 ± 0.4, *P*<0.01). In contrast, in Razinieh, the intensity of the sodium signal was lower in the vacuole than in the cytoplasm (20.8 ± 0.5 versus 38.0 ± 1.5; *P*<0.05), indicating that additional sodium was not sequestered in the expanding vacuole but was kept the cytoplasm. The pattern in the differentiation zone was again different (**Figure 7**). Here, both genotypes exhibited more signal in the vacuole than in the cytoplasm. For Della, the value was 20.5 ± 0.4 in the vacuole versus 9.5 ± 0.5 in the cytoplasm (*P*<0.01), for Razinieh a vacuolar signal of 37.4 ± 1.4 versus a cytoplasmic signal of 27.3 ± 1.3 was measured (*P*<0.05) (**Figure 7**). Overall, the sodium signal for the differentiation zone of Razinieh roots was almost a factor 2 higher than in Della.

We also assessed the development of the Casparian Strip in the endodermis of the differentiation zone because this hydrophobic barrier would reinforce the passage of ions through the membrane separating endodermis and central cylinder, i.e., also the ions entering through apoplastic water flow. Under control conditions, the Casparian Strip, although detectable, was not very pronounced, nor obviously different between Della and Razinieh (**Supplementary Figures S8A, C**). However, in response to salt stress, the entire endodermis along with the adjacent layer of the central cylinder displayed a strong signal (**Supplementary Figures S8A, C**). In Razinieh the entire radial wall seemed to be suberinised, while in Della the Casparian Strip appeared more localised. This pattern is just inverse to that seen for sodium transfer to the shoot (**Figure 1B**). This is consistent with the finding that the main difference in sodium partitioning between the genotypes was not seen in the differentiation zone (where the Casparian Strip is already present), but in the distal elongation zone (where the Casparian Strip is not yet developed).

### Transcripts reporting the ABA status are differentially regulated between the genotypes

To probe for the role of ABA, we measured steady-state transcript levels of selected abscisic-acid related genes after 1 h and 6 h of salt (100 mM NaCl) treatment. These included 9-cis-epoxycarotenoid dioxygenase (*SbNCED1*) encoding a key rate-limiting enzyme in ABA biosynthesis, Stress-Activated Protein Kinase1 *(SbSAPK1)* as readout for signalling, and *bZIP-TF-TRAB1-like* (basic region leucine zipper transcription factor) to monitor downstream responses.

**Figure 8:**
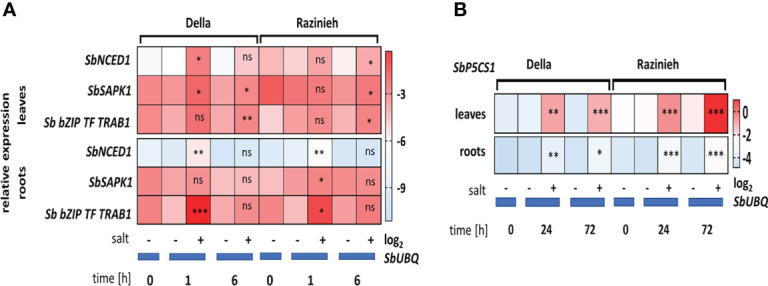
Heat maps representing the Log_2_ fold change of steady-state transcripts level of stress-related genes in response to salinity **(A)** *SbNCED1*, *SbSAPK1*, *bZIP-TF-TRAB1-like* and **(B)** *SbP5Cs1*. Thirteen days old Della and Razinieh seedlings were treated with 100 mM NaCl for 1, 6, 24 and 72 h. Values represent the mean of at least three independent biological replicates ± SE. Asterisks indicate a statistically significant difference between treatment and corresponding control, as determined by Student’s *t*-test (*P < 0.05, **p < 0.01, ***P < 0.001, and ns non-significant).

The transcripts for *SbNCED1* were induced more swiftly and to higher levels in Della as compared to Razinieh (**Figure 8A**). In the roots, transcript levels were induced up to 9-fold within the first hour in Della, but only by 3.5-fold in Razinieh, as compared to the controls. Also in the leaves, the induction in Della was more pronounced (around 10-fold within 1 hour of salt stress) than in Razinieh (around 2-fold). However, here, the ground levels of this transcript were much higher in Razinieh than in Della. For *SbSAPK1*, the regulation pattern for this transcript was inversed between roots and leaves, but at low amplitudes. But, *SbbZIP-TF-TRAB-1* most salient difference was in the roots.

### The key enzyme for proline synthesis is induced more strongly in Razinieh

We had seen that proline was accumulating in leaves of both genotypes in response to salinity (**Figure 3B**) (swifter and stronger in Della), while this response was not very pronounced in the roots. To get more insight into this adaptive response, we scored the transcripts for *SbP5Cs1* (D^1^-pyrroline-5-carboxylate synthase1), the first committed enzyme of the pathway, channelling glutamate towards proline biosynthesis (**Figures 8A, B** **)**. In roots, the induction of *SbP5Cs1* under salinity was weak, with a roughly 2-fold higher level in Razinieh over Della at both time points. In leaves, in contrast, the induction was very strong and clear, the steady-state levels at 72 h were significantly higher in Razinieh over those seen in Della. This is interesting, because proline levels at this time point (day 3) were lower in Razinieh as compared to Della (**Figure 3B**), although subsequently both genotypes reached a similar level at day 6 of the treatment (around 190- fold comparing to the corresponding controls) (**Figure 3B**). This accumulation seems to initiate earlier in Della, while in Razinieh it has to be compensated with some delay by a more pronounced expression of this key enzyme.

### The pattern of salt-induced jasmonate accumulation differs between the genotypes

**Figure 9:**
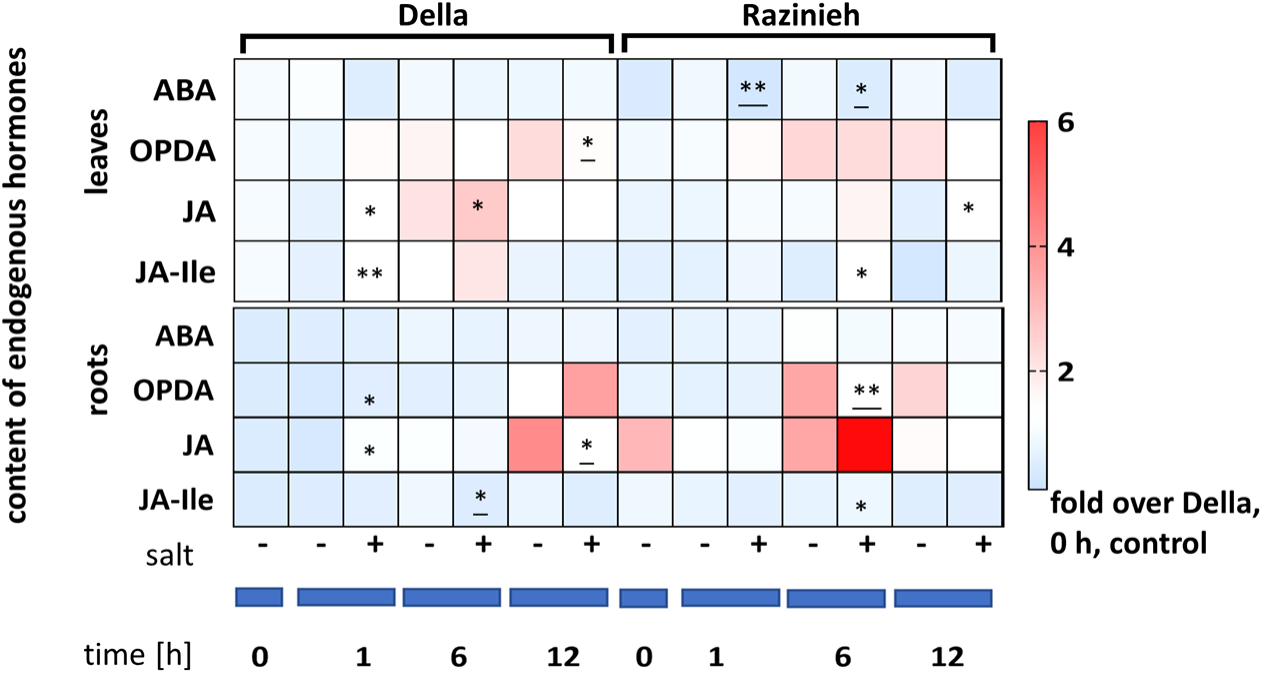
Heat maps representing the Log_2_ fold change of endogenous hormones content compared to the control of Della at day 0 in roots and 2^nd^ leaves of Della and Razinieh seedlings treated with 100 mM NaCl for 1, 6, and 12 h. Values represent the mean of at least three independent biological replicates ± SE. Asterisks indicate a statistically significant difference between treatment and corresponding control, as determined by Student’s *t*-test (*P < 0.05, **p < 0.01, and * significant decrease).

Since salinity induces a rapid response of jasmonates, often followed by a slower response of ABA, we followed the steady-state levels of ABA, OPDA, JA, and JA-Ile up to 12 h of salt treatment (**Figure 9**). The most salient difference was a strong and transient accumulation of JA in the ground level in Razinieh roots, it was 3-fold higher over that in Della. Although JA increased in Della by ~3-fold within 1 h of treatment compared to corresponding control, it did not even approximate the ground level seen in Razinieh. Interestingly, the response in the leaves was a mirror image. Here, in Della, JA accumulated in response to salinity, while the response was less pronounced in Razinieh. Also the changes of JA were accompanied by nearly equivalent increases in JA-Ile, indicating that JA was efficiently conjugated to JA-Ile. Compared to the fluctuations seen for the jasmonates, the steady-state levels of ABA were fairly constant, whereby the ground levels in the leaves were around 20% higher in Della than in Razinieh.

## Discussion

In the present work, we investigated the responses of a contrasting pair of sorghum genotypes to salt stress. Della, a sweet sorghum, is endowed with tolerance to salinity, while Razinieh, a grain sorghum, is more susceptible. Albeit, the phenotypic differences between sweet and grain sorghums, are useful for breeding, they are genetically closely related. According to previous population genetic studies, they are not discernible by molecular markers along racial subtypes (Morris et al., 2013; Bihmidine et al., 2015). We could show that the salt tolerance of Della is linked with a restrained translocation of sodium from root to shoot, supported by efficient vacuolar Na^+^ sequestration in the distal elongation zone as reported by a fluorescent sodium dye. In addition, the leaves of Della exhibited a more efficient redox homeostasis and accumulation of proline, while sucrose was more efficiently transferred to the roots, when the plants were challenged by salinity. Additional salient differences concerned the accumulation of glycine, the steady-state levels of jasmonic acid and its bioactive conjugate JA-Ile, and several transcripts, involved in the synthesis of ABA and proline. A given response to a stress could either be a manifestation of stress damage, or an expression of stress adaptation. It is difficult to tell the nature of a given event if this event was analysed in an isolated manner in a single genotype. However, when the observed difference can be compared over a contrasting pair of genotypes, it becomes possible to delineate stress adaption from stress damage. In the following, we discuss a working model, where, in the tolerant genotype Della, sodium sequestration in vacuoles of the root elongation zone along with a reduced sodium translocation to the shoot is accompanied by a more efficient translocation of sucrose from the shoot to the root. Both mechanisms sustain root growth under salinity and reduce ionic challenge to photosynthesis, such that the accumulation of proline as compatible osmolyte can proceed more swiftly. The resulting buffering against oxidative stress preserves photosynthetic efficiency, as indicated by reduced photorespiration monitored by lower glycine levels in Della. This model allows to propose metabolic and phytohormonal signatures of salt adaptation versus salt damage and to derive testable implications on the role of jasmonates as local and systemic signals that orchestrate the response to salt stress.

### Gaining time: Della can withhold sodium in the root more efficiently

Depending on duration and severity of salt stress, and on developmental state, the challenged plant needs to allocate resources to different strategies (impede ion influx, sequester sodium, induce necrosis, sustain growth). The superior performance of Della is linked with a more efficient exclusion of sodium from shoot and leaves, although comparable levels of sodium accumulate in the roots.

What are the causes behind this differential transfer of sodium to the shoot? A more efficient extrusion of sodium through the SOS1 exporter, or sequestration in the vacuole of root cortex cells? Our CoroNa Green data show clearly that the main difference between the genotypes occurs already in the distal elongation zone of the root, where Della is successfully accumulating sodium into the vacuole, while Razinieh fails to do this (**Figure 6**). This is supported by the clear induction of *SbNHX2* in roots of Razinieh under salt stress, indicative of a stronger requirement for this protein, while Della does not exhibit such an induction (**Figure 4A**). Instead, the differentiation zone looks fairly similar for the two genotypes. Thus, the symplastic route of uptake through the root hairs seems to be irrelevant in this context. Likewise, the development of a Casparian strip, while responsive to salt stress, can be ruled out as mechanism responsible for the differential transfer of sodium. Overall, sodium sequestration into the vacuole seems to play the main role for retaining sodium in the root, while sodium exclusion might only be a minor factor. This is congruent with conclusions on salinity responses in barley (Wu et al., 2019). The higher ground level of *HKT1* transcripts in the root of Della indicate that sodium retrieval from the xylem sap through HKT1 transporters (for review see Keisham et al., 2018) might contribute as well to some extent. Since potassium levels in the shoot persisted significantly better in Della, **a**ctivation of stelar potassium outward rectifiers (Maathuis, 2006), or of the K^+^ selective KUP/HAK/KT transporters might be involved as well (Han et al., 2016). In rice, knockout of OsHAK1 leads to salt susceptibility, while overexpression of this transporter helps the plants to cope with salt stress (Chen et al., 2015).

### Sugar helps against salt: Della can sustain sugar transport into the root under salt stress

As principal products of photosynthesis, sugars (mainly sucrose, glucose, and fructose) are particularly responsive to abiotic stress (for review see Lemoine et al., 2013), not only to provide the energy for adaptation, but also as compatible osmolytes, and signalling molecules for stress-responsive gene regulation (for review see Rosa et al., 2009). Sucrose accumulates more efficiently in the roots in Della, while in Razinieh, more sucrose remains in the leaves **(** **Figure 3A** **)**. Thus, the accumulation of sucrose in salt-stressed Della roots has to be interpreted as hallmark for successful adaptation. One would predict that transcripts of sucrose transporter genes would be upregulated in response to salinity, as it has been observed in rice under drought and salt stress (Mathan et al., 2020). This rapid accumulation of sucrose in the root of Della is accompanied by a very rapid and conspicuous accumulation of fructose and glucose in the leaves, a phenomenon that is not seen in Razinieh **(** **Figure 3A** **)**, consistent with data from perennial ryegrass with contrasting salt tolerance (Hu et al., 2013).

To sustain sucrose transport to the root as adaptive mechanism, photosynthesis must be sustained even in presence of sodium ions. However, the osmotic component causes stomatal closure, constraining the access of carbon dioxide, while, on the other hand, oxygen from water-splitting at photosystem II cannot be removed and accumulates, such that RubisCO progressively acts as decarboxylase leading to increased photorespiration (Munns and Tester, 2008). Photorespiration leads to the breakdown of Ribulose-1,5-bisphosphate into phosphoglycerate and phosphoglycolate. Phosphoglycolate is transported into the peroxisome and converted to glyoxylate by the glycolate oxidase, which is then reacting with glutamate into glycine and a-ketoglutarate (for review see Bauwe et al., 2010). Thus, accumulation of glycine at concomitant depletion of glutamate can be used as readout of photorespiration. Our metabolomic profile of amino acids shows that this signature is developing in leaves of Razinieh more rapidly and at higher amplitude (**Figure 3B**). As C_4_ crop, sorghum can use malate to store carbon dioxide and to release it on demand by virtue of the NADP-Malate Dehydrogenase (for review see Yang et al., 2020). This enzyme seems to promote salinity tolerance (Sui et al., 2015; Yang and Guo, 2018). Whether the more efficient quenching of photorespiration in Della is linked with a higher expression of this enzyme, is a testable implication of this hypothesis.

The higher photorespiration rate will impair photosynthetic efficiency and, thus, the ability to transfer sucrose into the root for the compensation of salinity. It will, however, also cause the accumulation of reactive oxygen species: As a consequence of reduced carbon fixation, reduced NADPH will accumulate, feeding back on electron transport, causing a so-called hyperreduction (Allakhverdiev et al., 2000). The overshoot of electrons is transferred to molecular oxygen, generating ROS. This can happen at photosystem II (for instance through cytochrome b_559_), at photosystem I (through a Mehler reaction), or at the plastoquinone pool (Khorobrykh et al., 2015). In addition, the conversion of glycolate to glyoxylate in the peroxisome is using molecular oxygen as acceptor, generating hydrogen peroxide as additional ROS (Bauwe et al., 2010).

Thus, the superior performance of Della roots to cope with salt stress is linked with sucrose transfer to the root, which in turn depends on the suppression of photorespiration under salinity, and the vigour of redox homeostasis.

### Della protects photosynthesis by bolstering redox homeostasis against salt stress

In fact, there are indications for a more robust redox homeostasis in Della, such as reduced MDA levels. The rapid increase in SOD activity and of APX indicate that superoxide radicals produced at PSI are converted to hydrogen peroxide, and then dissipated by a membrane-bound thylakoid ascorbate peroxidase, which is not the case in Razinieh. The strong activity of SOD is followed by a concomitant slight increase of peroxide steady-state levels that are rapidly mitigated though (**Figure 2B**). The supportive role of plastidic SOD and of APX for salt tolerance has also been found in Brassica (Tseng et al., 2007; Saxena et al., 2020). In contrast, the more pronounced photorespiration in Razinieh requires peroxisomal CAT to scavenge the H_2_O_2_ released during glycolate oxidation (Apel and Hirt, 2004; Miller et al., 2010). In fact, we see a stronger early increase of CAT activity, which is collapsing later, linked with a more pronounced increase of peroxide steady-state levels and of MDA as damage readout. Thus, the hydrogen peroxide accumulation seems to stem from different functional contexts in the two genotypes. The late rise in GR and POD activities in Razinieh **(** **Figure 2C** **)** have to be interpreted as stress markers rather than as hallmarks of adaptation. GR is a key enzyme in the ascorbate glutathione cycle (Noctor et al., 2002; Ahmad et al., 2010). The same holds true for the late increase in POD activity in Razinieh (**Figure 2C**), correlating with a strong accumulation of phenolic compounds (**Supplementary Figure S5A**). Especially, vacuolar POD may be relevant here, which kicks in when scavenging by the ascorbate-glutathion cycle in the chloroplast is not sufficient, such that H_2_O_2_ escapes to the vacuole (Jovanović et al., 2018). In Della, the higher level of non-enzymatic antioxidants as evident from the DPPH and ABTS free radical scavenging assays **(** **Supplementary Figures S6A, B**, and **Figures 2F, G** **)** might render the late activation of CAT, POD, and GR partially dispensable, which is certainly the more resource-efficient approach as compared to synthesis of these enzymes.

### Proline accumulation plays a significant role in adaption to salt stress

Accumulation of proline as compatible osmolyte allows for osmoregulation, but also protects the photosynthetic apparatus against ROS (for reviews see Szabados and Savouré, 2010; Verslues and Sharma, 2010). Under abiotic stress, proline is mainly generated from glutamate utilising NADPH as reducing agent catalysed by 1-pyrroline-5-carboxylate synthetase (P5CS) as rate-limiting enzyme (Hare and Cress, 1997). The increase in proline content in the leaves at concomitant reduction of glutamate (**Figure 3B**) indicates that this pathway is activated in response to salt stress. This increase is sustained more vigorously in Della, especially during the decision phase in the root, where the initial salt content is kept restrained at the level attained during the first day. The prolonged induction of transcripts for the rate-limiting enzyme, *SbP5Cs1*, do not come with a corresponding increase in proline steady-state levels in Razinieh. Since proline can dissipate both, superoxide, and hydrogen peroxide, giving rise to hydroxyproline (for review see Liang et al., 2013), indicates that proline is consumed by ROS scavenging, such that the initial pool is dissipated earlier than in Della with the consequence that *SbP5Cs1* needs to remain activated even after day 1. Sustained activity of this rate-limiting enzyme must, therefore, be interpreted as marker for stress damage, not as marker for stress adaptation. A testable implication of this hypothesis would be that hydroxyproline levels in Razinieh are expected to be higher than those in Della. In contrast to *SbP5Cs1* transcripts, steady-state levels of proline, the product of this enzyme, can be used as readout for tolerance, in line with data on rice, where higher proline steady state levels in the wild species *O. australiensis* correlated with a higher salt tolerance (Nguyen et al., 2021). The higher proline levels in Della might also be the mechanism behind the superior Relative Water Content in Della leaves under stress (**Supplementary Figure S3D**), because higher proline levels should lower the water potential in the stressed leaves (Sharma and Verslues, 2010), such that they remain turgescent.

An interesting detail is the difference in timing. The accumulation of proline is more pronounced and sustained in Della, although the translocation of sodium ions to the shoot was slower. If *SbP5Cs1* would respond to the local level of sodium, the opposite would have been expected.

### Adaptation or susceptibility: a matter of systemic signalling?

If the adaptation of leaves to salinity is more efficient in Della because it initiates more swiftly, and if this response precedes the arrival of the sodium front through the transpiration stream, there must be a rapid systemic signal that is deployed in the challenged root. This signal is unknown, but it is noteworthy that jasmonic acid (JA) and its bioactive conjugate JA-Ile, are already significantly elevated one hour after the onset of salt stress. Also in the root, JA responds early, but here no significant increase of JA-Ile is observed (**Figure 9**). If JA is not converted to JA-Ile, it might be converted to the volatile MeJA, which could signal to the leaves that the roots are under challenge, such that anticipative responses (such as the activation of proline synthesis) can be deployed. A similar systemic signal had been proposed for G-protein dependent inhibition of cell division in the leaves of rice and maize challenged by salinity (Urano et al., 2014). While the role of MeJA for salt tolerance has been studied intensively (for a recent review see Delgado et al., 2021), the aspect of its relevance for systemic signalling seems to be vastly neglected.

**Figure 10:**
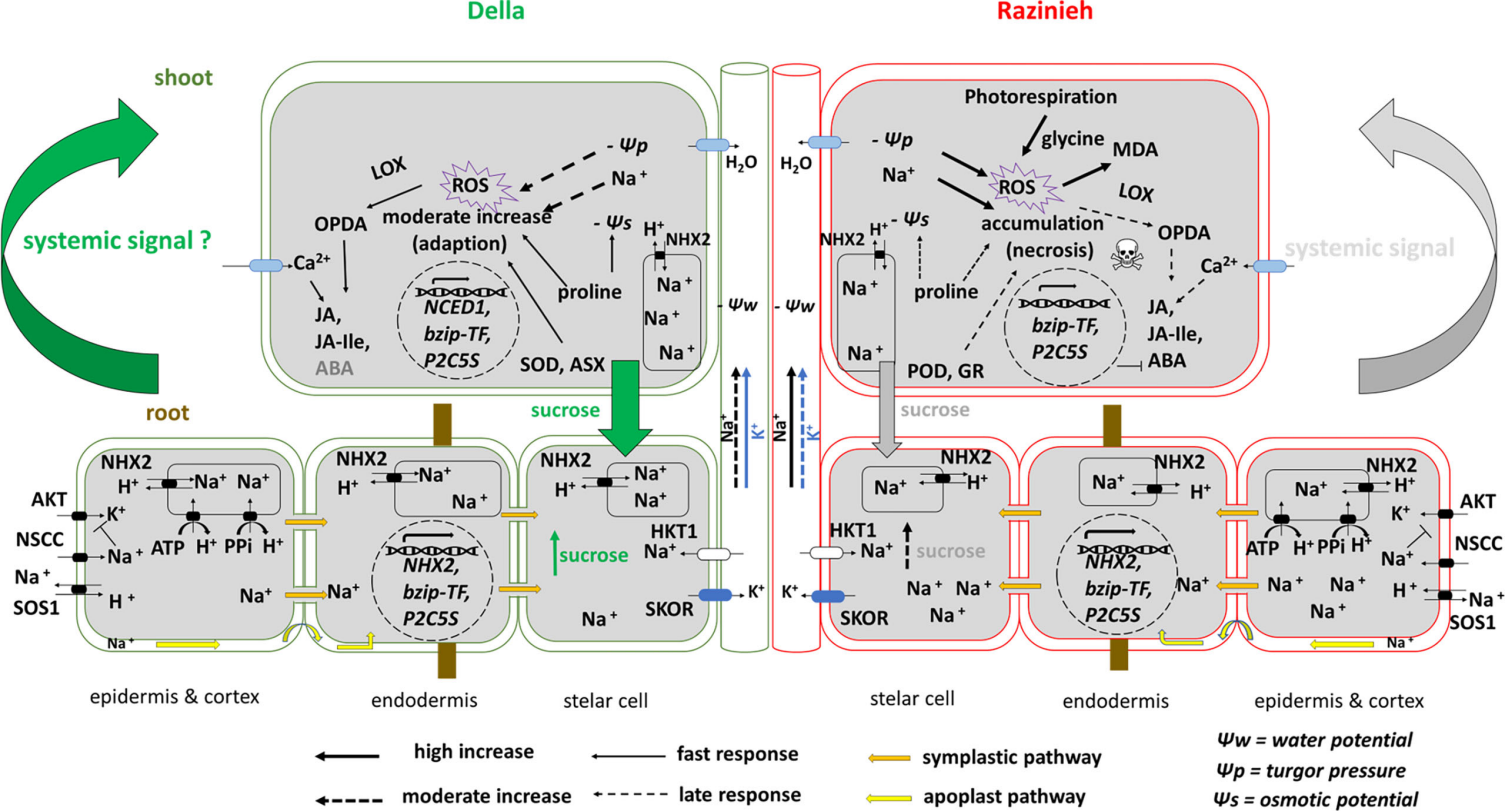
Visual model representing two contrasting sorghum genotypes to salt stress. The salt adaption in Della genotype is linked with better sequestration of Na^+^ in the vacuoles of root cortex cells and restricted translocation of Na^+^ from root to shoot. In addition to rapid translocation of sucrose to the roots and depending on efficient redox homeostasis and proline accumulation in leaves. Meanwhile glycine accumulation in Razinieh leaves and H_2_O_2_ abundance were considered as markers of high photorespiration rate coupled with the observed phenotype of leaf necrosis related to stress susceptibility which could be linked to the accumulation of MDA as a result of higher lipid peroxidation due to ROS accumulation.

Our study supports a model (**Figure 10**) where the swift systemic signal released by Della roots might be the central factor that renders Della salt tolerant. This rapid, systemic signal (that might be a jasmonate-related compound) allows for anticipative adaptation to the ionic stress that will arrive later. This adaptation (including activation of proline synthesis and ROS-scavenging enzymes) helps to buffer photosynthesis against sodium toxicity such that photorespiration is mitigated. The more efficient photosynthesis, in turn, allows to transfer sucrose into the root, such that the root can command the energy needed to sequester sodium ions in the vacuole of the distal elongation zone, which, in turn, slows down the transfer of sodium to the shoot, such that the shoot gains further time for adaptive measures. To what extent this self-amplifying functional circuit is later stabilised by activation of ABA signalling remains to be elucidated. The rapid activation of *SbNCED1* transcripts points into that direction, even though they do not seem to result in significant accumulation of ABA, at least during the time points considered in the current study. ABA signalling might act as stabiliser rather than as mediator of adaptation. A testable implication of our model would be, whether the two genotypes differ with respect to the temporal regulation of jasmonic acid methyltransferase. Overall, we think that the question of susceptibility versus tolerance is mainly a question of timing which is well in line with other cases, where salt stress has been studied not only with respect to molecular genetics, but with respect to its temporal dynamics (for review see Ismail et al., 2014a). This perspective shifts early signalling into the centre of interest for future studies.

## Data availability statement

The original contributions presented in the study are included in the article/**Supplementary Material**. Further inquiries can be directed to the corresponding author.

## Author contributions

EA designed the research, performed all experiments, and wrote the manuscript. AK, MR offered supervision and technical assistance. MR, BJ carried out metabolites analysis (HPLC-FLD and GC-MS). EE hosted minerals analysis (ICP-OES). BH hosted and supervised hormones analysis (UPLC-MS/MS). PN supervised the research, edited and complemented the manuscript. All authors contributed to the article and approved the submitted version.

## Funding

This work was supported by a full PhD scholarship from the Ministry of Higher Education of the Arab Republic of Egypt to EA.

## Acknowledgments

The authors acknowledge Dr. Gabriele Jürges for providing protocols for fixation and staining. We acknowledge support by the KIT-Publication Fund of the Karlsruhe Institute of Technology.

## Conflict of interest

The authors declare that the research was conducted in the absence of any commercial or financial relationships that could be construed as a potential conflict of interest.

## Publisher’s note

All claims expressed in this article are solely those of the authors and do not necessarily represent those of their affiliated organizations, or those of the publisher, the editors and the reviewers. Any product that may be evaluated in this article, or claim that may be made by its manufacturer, is not guaranteed or endorsed by the publisher.

## Abbreviations

ABA, Abscisic acid; AKT, Potassium channel; ABTS, 2,2′-azino-bis(3-ethylbenzothiazoline-6-sulfonate); ASX, Ascorbate peroxidase; BHA, Butylated hydroxyanisole; bZIP-TF-TRAB1-like, basic region leucine zipper transcription factor; CAT, Catalase; DPPH, 2,2-dipheny-1 -picrylhydrazyl; GR, Glutathion Reductase; H_2_O_2_, hydrogen peroxide; HKT1, High-affinity potassium transporter 1; JA, Jasmonic acid; JA-Ile, Jasmonate Isoleucine; MDA, Malondialdehyde; NCED1, 9-cis-epoxycarotenoid dioxygenase 1; NHX2, Na+/H+ vacuolar antiporter 2; NSCC, Non-selective cation channel; OPDA, 12-oxophytodienoic acid; P5Cs1, D1-pyrroline-5-carboxylate synthase 1; POD, Peroxidases; SAPK1, Stress-Activated Protein Kinase 1; SKOR, shaker-like K^+^ outward-rectifying channel; SOD, superoxide dismutase; SOS1, Salt Overly Sensitive 1; Ψw, Water potential; Ψp, Turgor pressure; Ψs, Osmotic potential.
