# Supplementary material for: Gain time to adapt: How sorghum acquires tolerance to salinity
Source: Front Plant Sci. 2022 Oct 17;13:1008172. doi: 10.3389/fpls.2022.1008172 (PMC9619063; doi:10.3389/fpls.2022.1008172)
Supplement: Supplementary file 1 [file DataSheet_1.zip › Supplementary materials and methods.DOCX]

Supplementary Material

**Supplementary methods:**

**Determination of soluble sugars by GC-MS.** Aliquots of 10 μl of the filtered extract were dried in a vacuum concentrator (Christ, Germany) without heating for 45 minutes. The tissue samples along with the respective reference standards for glucose, fructose, and sucrose (Sigma, USA) were derivatised (PAL, Chromtech evolution). Thirty microliters of methoxamine hydrochloride (20 mg/ml in pyridine) were added to these samples, and the mixture shaken for 60 min at 45°C. Subsequently, 45 μl of BSTFA (N,O-Bis(Trimethylsilyl)trifluoroacetamide) were added and the samples shaken again for 120 min at 45°C. The derivatised samples were injected at 250°C in a splitless mode with a helium gas flow set to 1 ml^.^min^-1^. Chromatography was performed with a 30-m Zebron Capillary GC-Column (ZB-5MS plus-Phenomenex, 30 m, 0.25 mm, 0.25 μm). All the samples along with the respective sugar standards were analysed by GC-MS (GC/MS/MS Agilent 7890A / 5975C / Chromtech Evolution 3, Agilent, Santa Clara, USA). The temperature program was set to an initial temperature of 60°C, followed by a linear ramp of 10°C^.^min^-1^ to 180°C and held at this temperature for 8 minutes. This was followed up by another linear ramp with a slope of 10°C^.^min^-1^ to 325°C and kept at this temperature for additional 3 minutes. Throughout the entire run, the transfer line was set to 290°C, the source to 230°C, and the quadrupole to 150°C. The raw data were processed by the Mass Hunter Qualitative Analysis software (Agilent, B.07.00) and the identification of the chromatographic peaks were validated using the mass spectra library NIST 14 (National Institute of Standards and Technology), along with the data from the sugar standards. For sugar calibration and quantification, reference standards for all three sugars were measured in triplicates for 8 different concentrations. The standard curve was then used to calibrate the sugar concentrations in the respective plant samples. These standards were also used for in-batch and inter-batch correction of the data analysis. Peak areas were normalised to the respective sugar standards and the dry weight of the samples used for extraction.

**Determination of total free amino acids using HPLC-FLD**. Total free amino acids were separated by HPLC (Agilent 1260 Infinity II) comprising a quaternary pump, an autosampler, a degasser, and a fluorescence detector. Online automated OPA/FMOC based derivatisation for the amino-acid standards, as well as the plant-tissue samples, was performed using the autosampler of the Agilent 1260 Infinity II system. Amino acids were separated on a Poroshell HPH-C18 column (4.6 × 100 mm, 2.7 μm) maintained at 40°C, using the binary gradient mobile phase with the same pre-set specifications as in the 1260 Infinity II amino acid solution system. In brief, prior to every injection, the column was equilibrated for 2 min, and 0.5 µl of the derivatised sample were injected. The pump flow rate was set to 2.0 mL/min with mobile phases A (10 mM Na_2_HPO_4_, 10 mM Na_2_B_4_O_7_, pH 8.2, 5 mM NaN_3_) and B (acetonitrile, methanol, and deionised water in volumetric ratio of 45:45:10). The applied linear gradient was set to 0–0.25 min 2% B, 9.40 min 53% B and 9.50 min 100% B. The reference standards for the 21 amino acids were measured in triplicates for 5 different concentrations for each batch of the HPLC run. Data acquisition and analysis were performed with OpenLAB CDS ChemStation Edition software (Agilent, Santa Clara, CA, USA). The obtained standard curve was then used to calibrate the amino-acid concentrations in the respective plant samples. These standards were also used for in-batch and inter-batch correction of the data. Peak areas were normalised for the respective amino acid standards and the dry weight of the samples used for extraction.

# Supplementary Figures legends

**Supplementary figure S1.** Mathematical model representing the coefficient for sodium transfer from root to shoot.

**Supplementary figure S2.** Content of potassium ions in (a) shoots and (b) roots of sorghum genotypes Della and Razinieh (c) K/Na ratio in the shoots and (d) K/Na ratio in the roots. Thirteen days old Della (white bars), and Razinieh (grey bars) seedlings were stressed in aqueous NaCl (100 mM) solution and collected after 1, 3, 6, 9, and 12 days. Values represent the mean of at least three independent experiments ±SE. Different letters show significant differences between different genotypes and treatments according to Duncan’s test (*P*<0.05). Asterisks indicate a statistically significant difference between genotypes, as determined by Student’s *t*-test (**P*< 0.05, and ***p*<0.01).

**Supplementary figure S3.** Effects of salt stress on the 2^nd^ leaf phenotyping of Della and Razinieh seedlings. (a) The second leaf of thirteen-days-old sorghum seedlings subjected to a 100 mM NaCl solution for 6 days. (b) The percentage of second leaf green area under stress compared to each corresponding control in thirteen-days-old sorghum seedlings treated with 100 mM NaCl solution for 1, 3, and 6 days (c) chlorophyll (a+b) content of second leaf in thirteen-days-old sorghum seedlings subjected to a 100 mM NaCl solution for 1, 3, and 6 days (d) Second leaf RWC% in thirteen-days-old sorghum seedlings which were subjected to a 100 mM NaCl solution for 1, 3, and 6 days. Values represent the mean of at least three independent replicates ±SE. Different letters show significant differences between different genotypes and treatments according to Duncan’s test (*P*<0.05). Asterisks indicate a statistically significant difference between genotypes, as determined by Student’s *t*-test (***p*<0.01).

**Supplementary figure S4.** The dry weight (%) of shoot (a) and root (b) under stress compared to each corresponding control of Della and Razinieh thirteen-days-old seedlings subjected to a 100 mM NaCl solution for 1, 3, 6, 9, and 12 days.  Values represent the mean of at least five independent experiments ±SE. Asterisks indicate a statistically significant difference between genotypes, as determined by Student’s *t*-test (**P*< 0.05, and ***p*<0.01).

**Supplementary figure S5. (**a) The level of total polyphenolics contents, and (b) the level of total flavonoids contents in thirteen-days-old seedlings shoots treated with 100 mM NaCl solution for 1, 3, and 6 days. Values represent the mean of at least three independent experiments ±SE. Different letters show significant differences between different genotypes and treatments according to Duncan’s test (*P*<0.05).

**Supplementary figure S6**. The scavenging activity of methanolic extract of sorghum seedlings shoots treated with 100 mM NaCl for 6 days against (a) the synthetic free radicals 2, 2-diphenyl-1 - picrylhydrazyl (DPPH) and (b) 2,2′-azino-bis(3-ethylbenzothiazoline-6-sulfonate (ABTS) using Butylated hydroxyanisole (BHA) as internal standard. Values represent the mean of at least three independent biological replicates ±SE.

**Supplementary figure S7.** The steady-state transcripts level of salt stress-related genes (a) *SbSOS1* in 2^nd^ leaf, (b) *SbSOS1* in roots*,* and (c) *SbNHX2* in 2^nd^ leaf. Seedlings were treated with 100 mM NaCl for 1 and 6 h. Values represent the mean of at least three independent biological samples ±SE. Different letters show significant differences between different genotypes and treatments according to Duncan’s test (*P*<0.05).

**Supplementary figure S8.** Visualisation of Casparian strips development under salt stress. 15 µm thick cross sections were cut by a microtome from paraffin-fixed adventitious roots of thirteen-days-old seedlings subjected to a 100 mM NaCl solution for 6 days and the sections were stained with berberine aniline blue and viewed using blue light. Arrows show Casparian strips in the endodermis (en). (a) control Della, (b) stressed Della, (c) control Razinieh, and (d) stressed Razinieh.

**Supplementary Table S1.** The sequences of forward and reverse primers for the genes
of interest and the housekeeping gene Ubiquitin (used for normalization).
