# Supplementary figures and images for: Gain time to adapt: How sorghum acquires tolerance to salinity
Source: Front Plant Sci. 2022 Oct 17;13:1008172. doi: 10.3389/fpls.2022.1008172 (PMC9619063; doi:10.3389/fpls.2022.1008172)

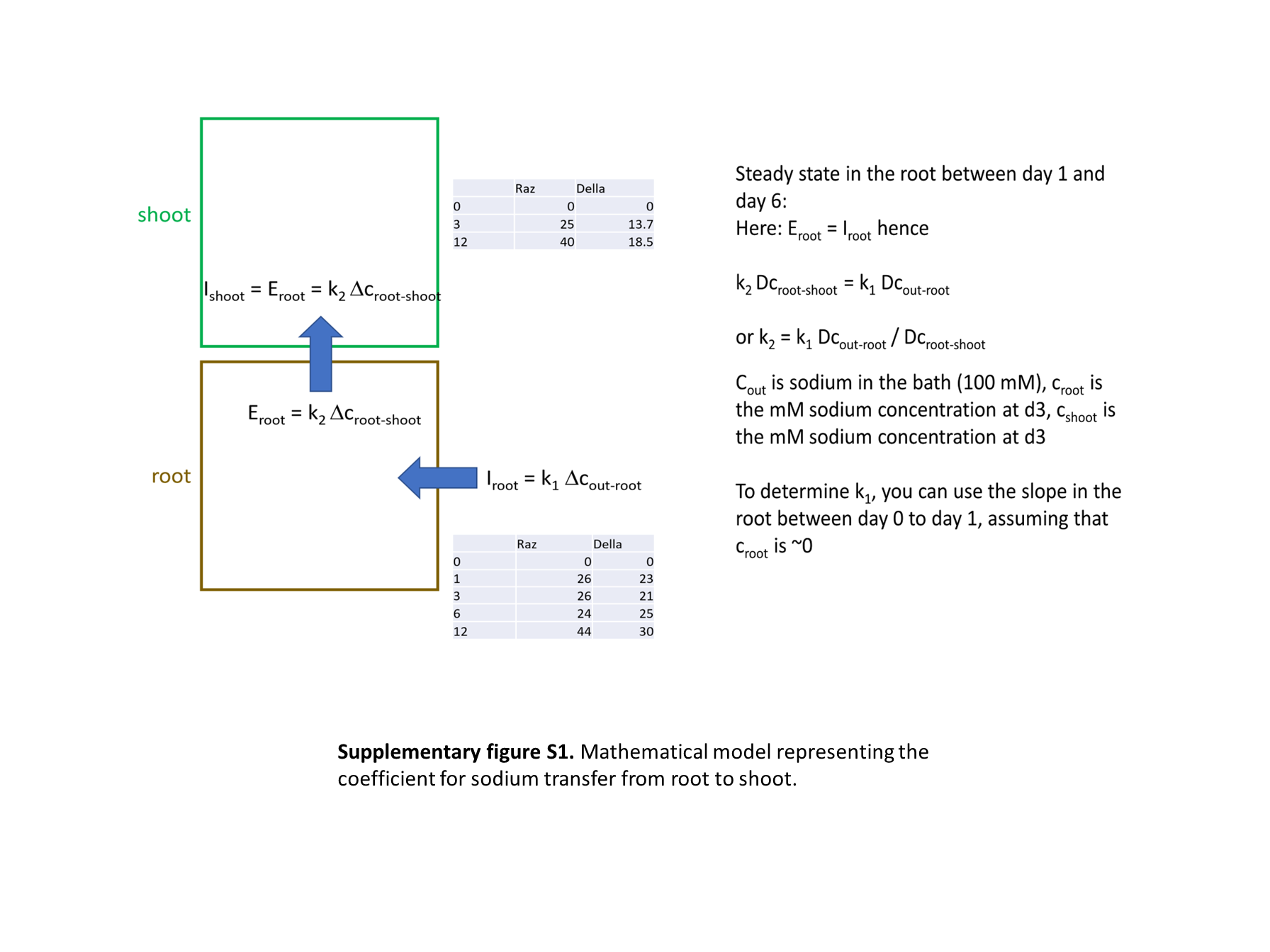

Supplement: Supplementary file 2 [file Image_1.tif]

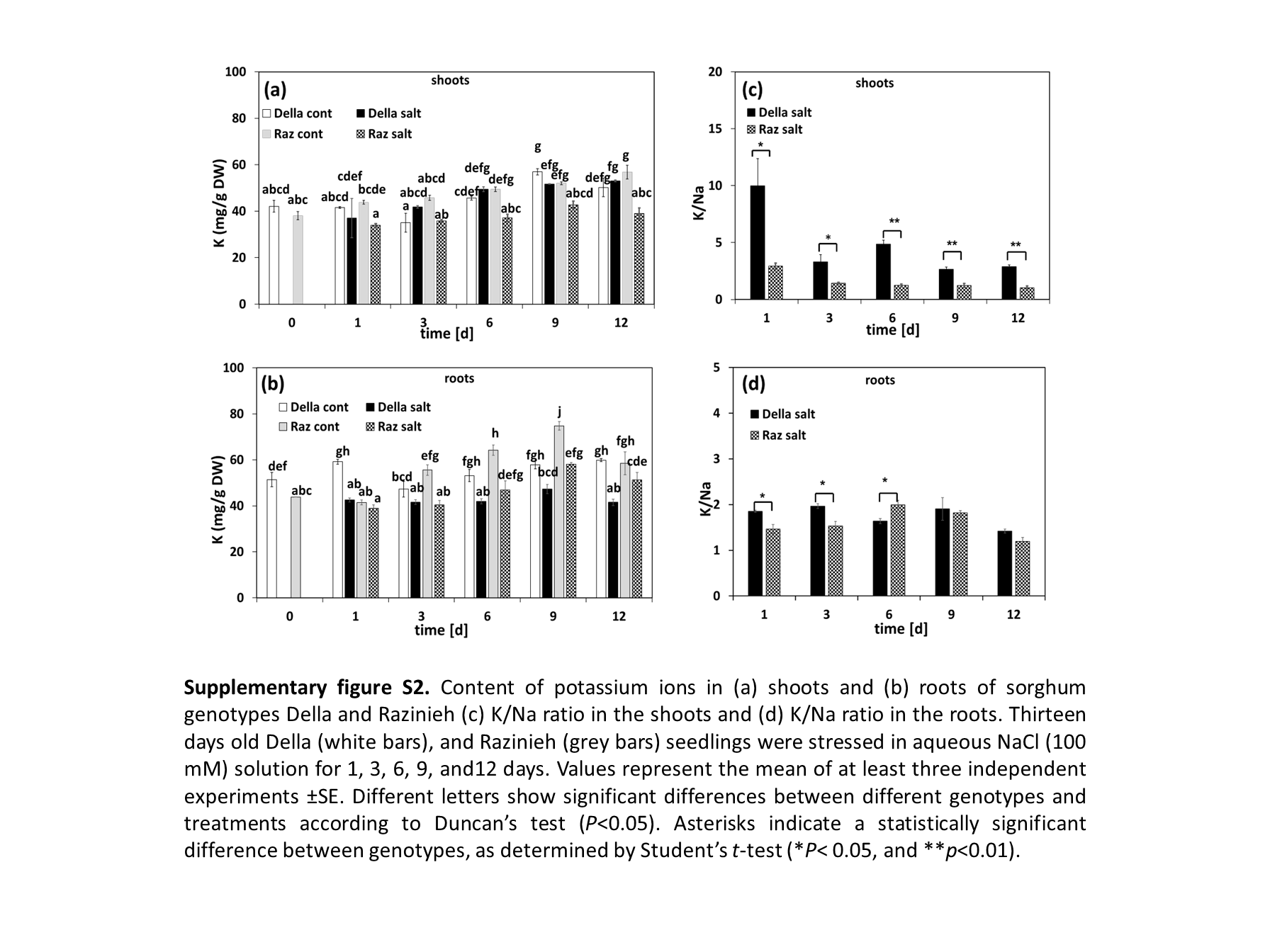

Supplement: Supplementary file 3 [file Image_2.tif]

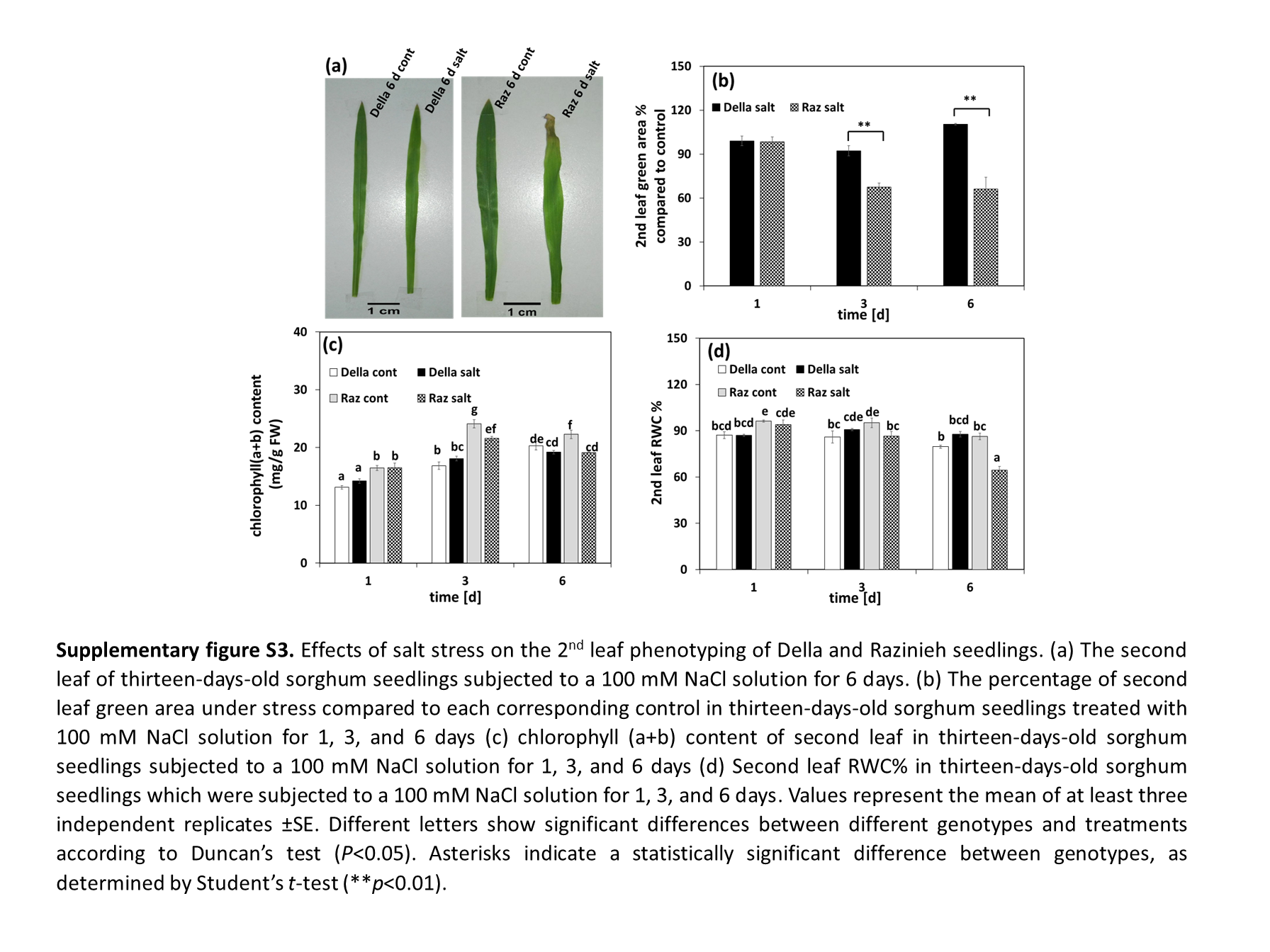

Supplement: Supplementary file 4 [file Image_3.tif]

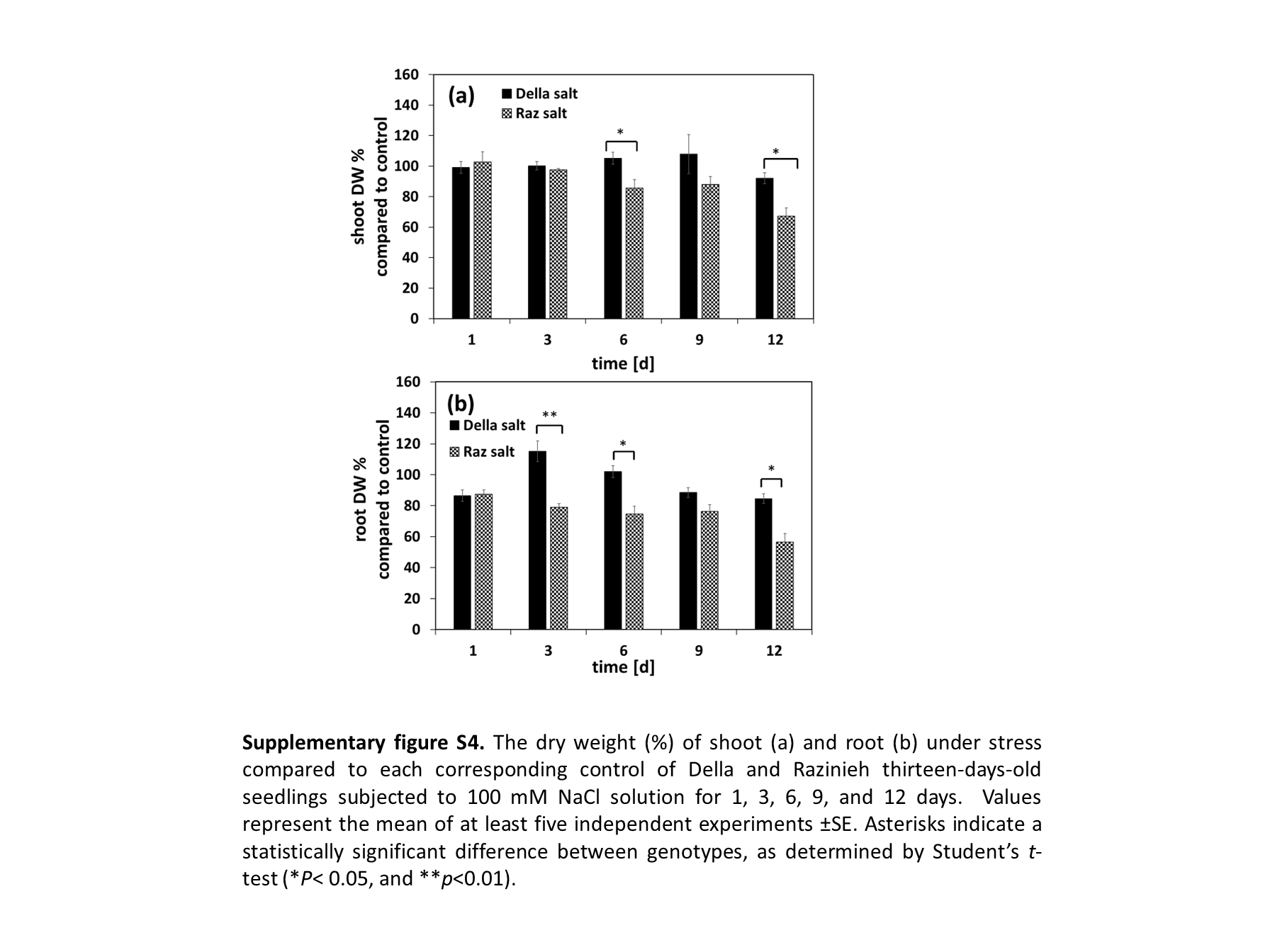

Supplement: Supplementary file 5 [file Image_4.tif]

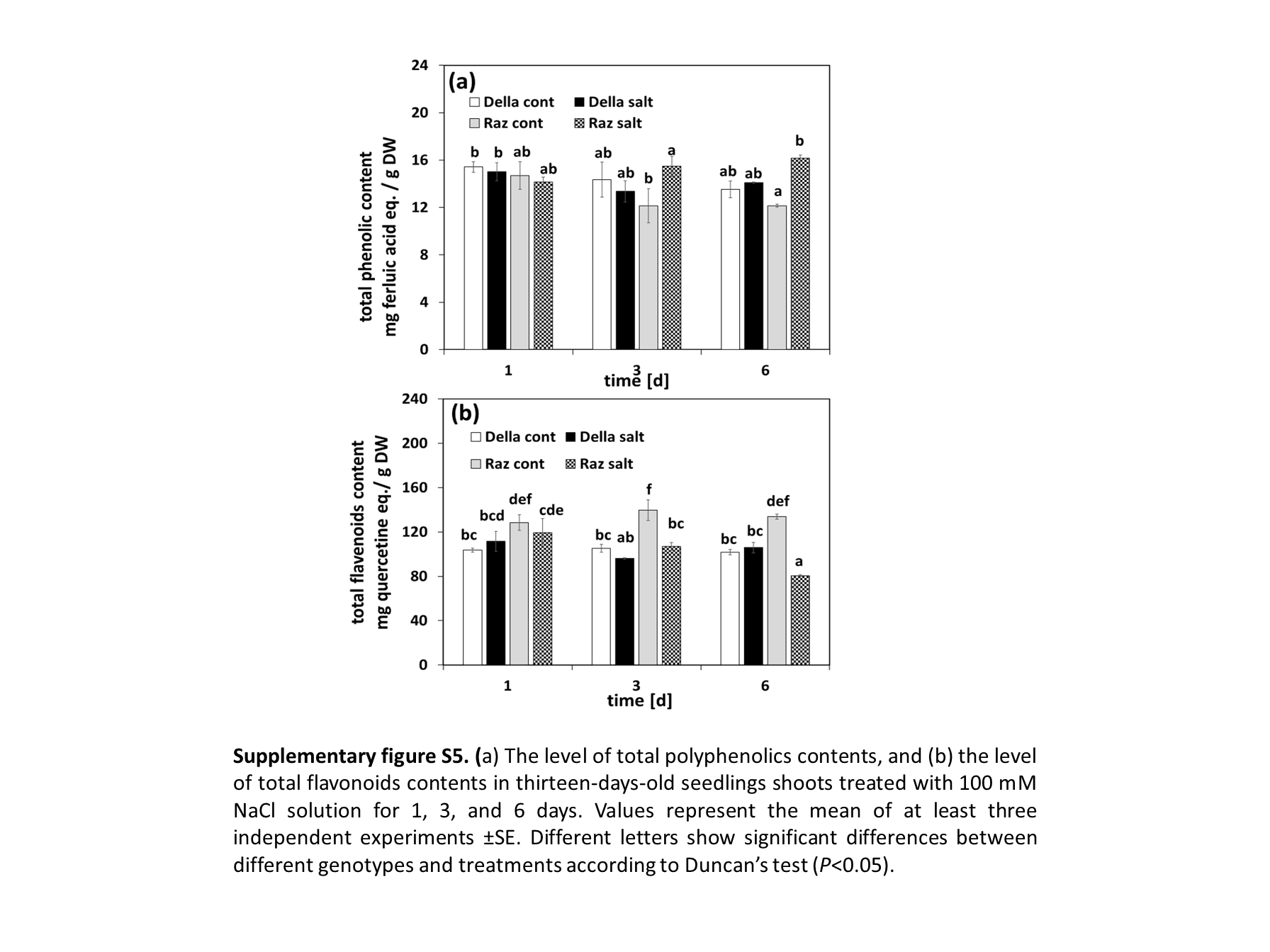

Supplement: Supplementary file 6 [file Image_5.tif]

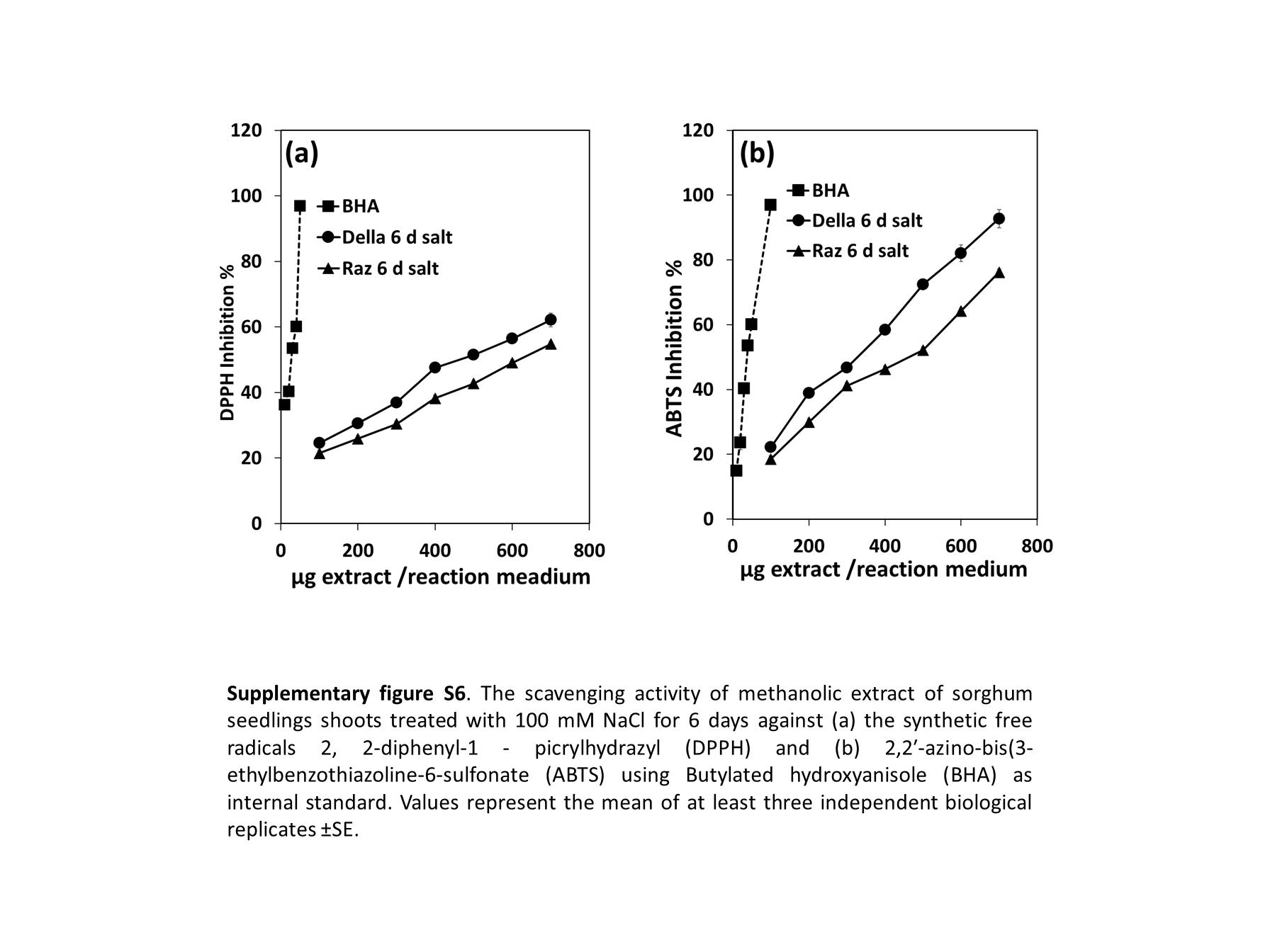

Supplement: Supplementary file 7 [file Image_6.tif]

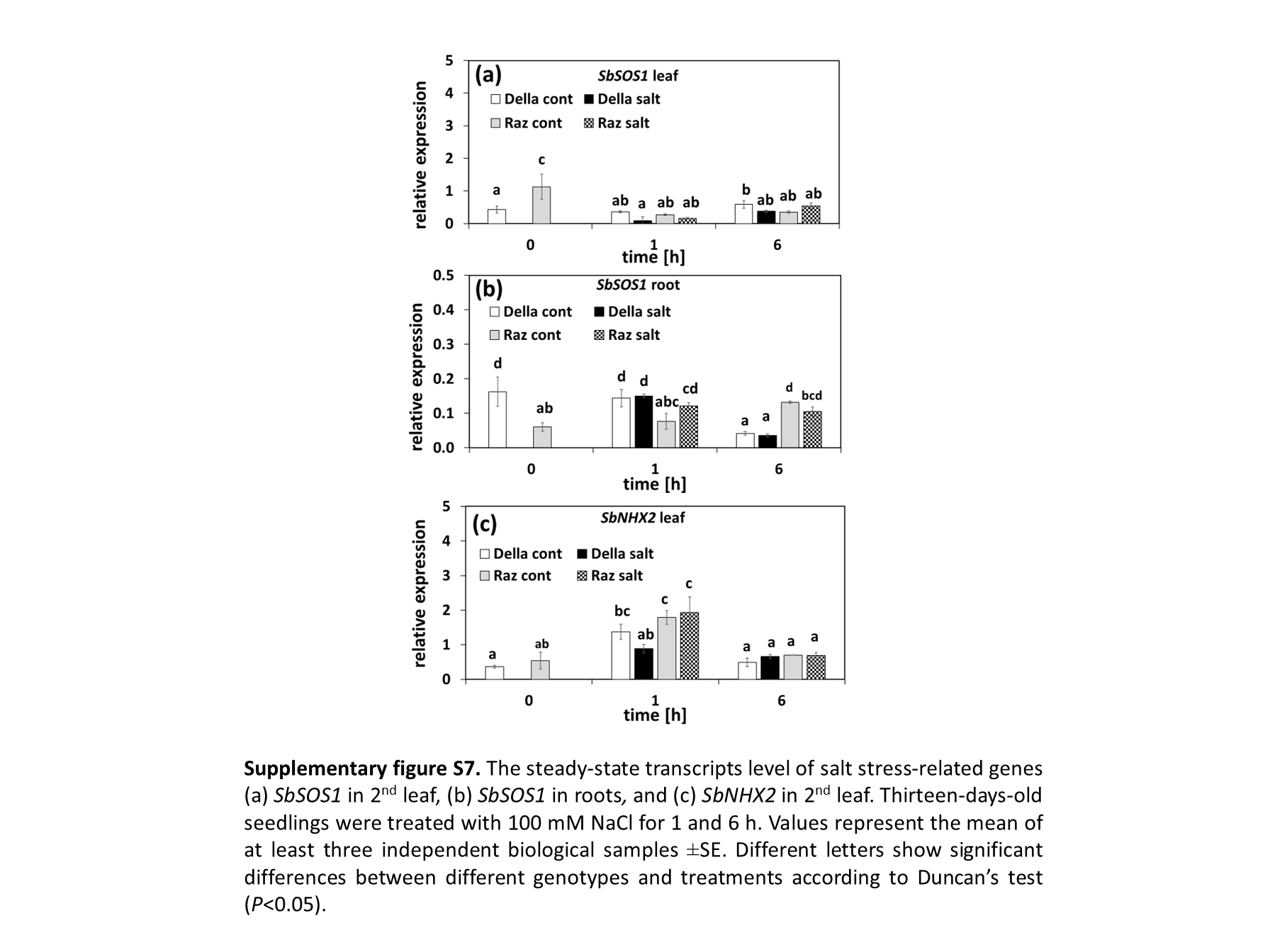

Supplement: Supplementary file 8 [file Image_7.tif]

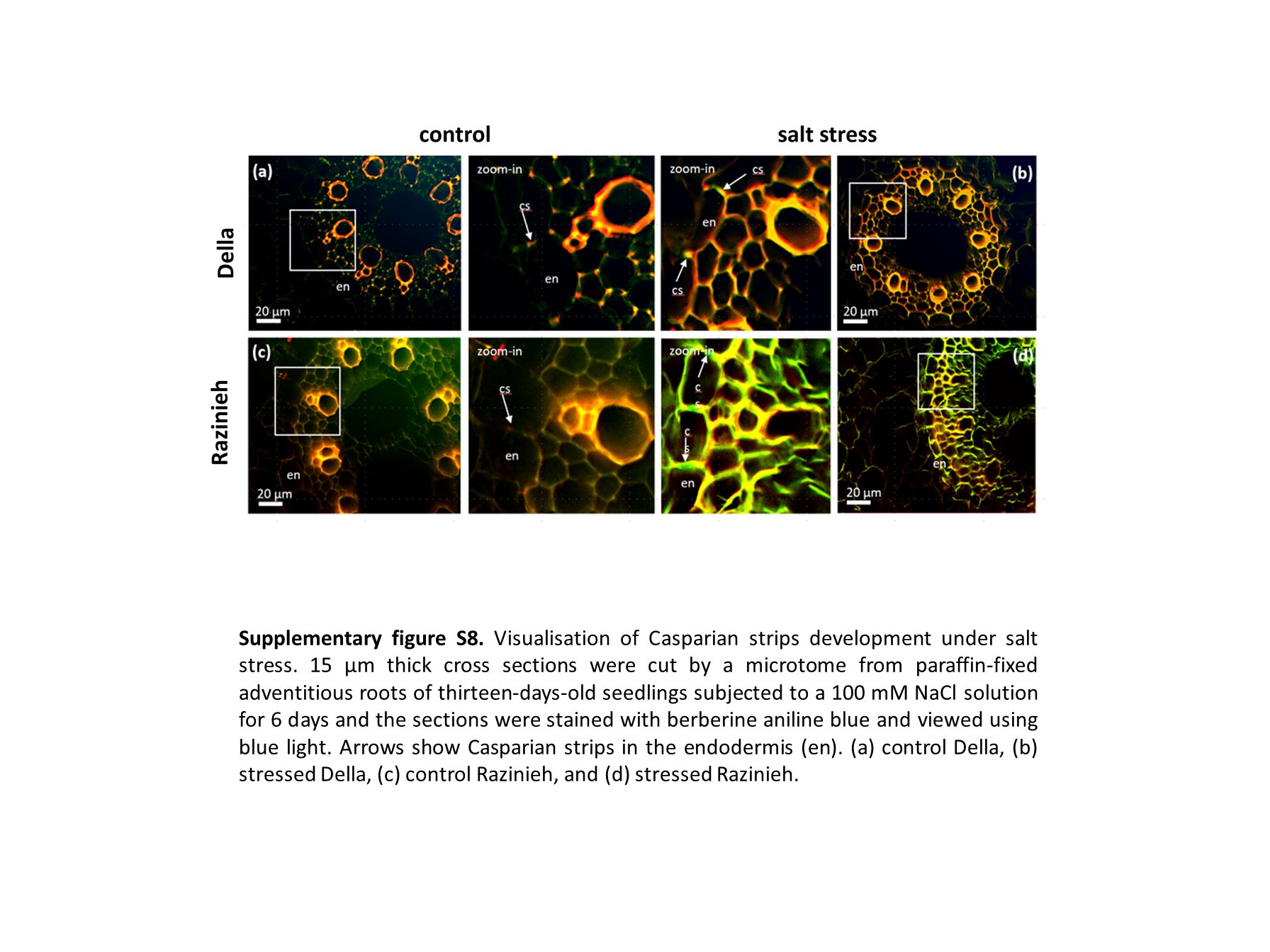

Supplement: Supplementary file 9 [file Image_8.tif]

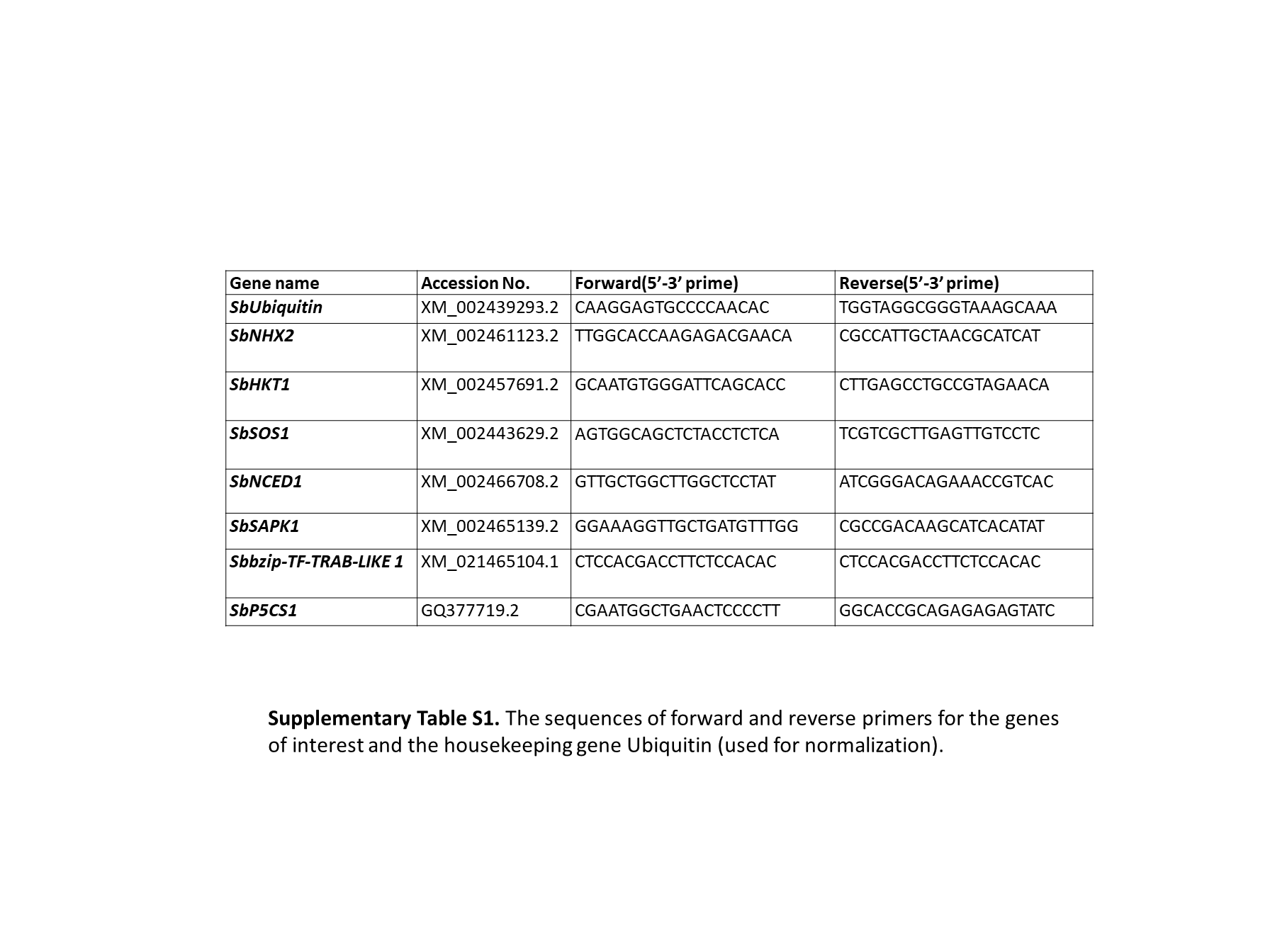

Supplement: Supplementary file 10 [file Image_9.tif]
